# Network analysis of association and clustering of adverse experiences, mental health symptoms and help-seeking among adolescents and adults in Ghana

**DOI:** 10.1136/bmjopen-2026-121157

**Published:** 2026-11-09

**Authors:** Victoria Awortwe, Meena Daivadanam, Samuel Adjorlolo, Joanne Woodford, Erik Martin Gustaf Olsson, Matteo Magnani

**Affiliations:** 1 CIRCLE- Complex Intervention Research in Health and Care, Department of Women's and Children’s Health, Uppsala University, Uppsala, Sweden; 2 International Child Health and Nutrition, Department of Women’s and Children’s Health, Uppsala University, Uppsala, Sweden; 3 Department of Mental Health Nursing, University of Ghana, Legon, Ghana; 4 Research in Cardiovascular Psychology, Department of Women’s and Children’s Health, Uppsala University, Uppsala, Sweden; 5 Department of Information Technology, Uppsala University, Uppsala, Sweden

**Keywords:** adverse experiences, mental health symptoms, help-seeking, adolescents, adults, network analysis, Ghana

## Abstract

**Abstract:**

**Objectives:**

To examine the (1) conditional associations between adverse experiences, mental health symptoms and help-seeking, represented as nodes within the adolescent and adult networks, including gender-specific associations, (2) centrality of nodes within the adolescent and adult networks and (3) clustering of adverse experiences, mental health symptoms and help-seeking among adolescents and adults in Ghana.

**Design:**

Cross-sectional study.

**Setting:**

This study was conducted in three senior high schools located in three regions in Ghana, from June-September 2019.

**Participants:**

A total of 1884 participants were included in this study, of which 1160 were adolescents aged 12–17 years and 724 were adults aged 18–45 years.

**Primary and secondary outcome measures:**

Networks for adolescents and adults were estimated using Ising models, and assessed for sensitivity and stability. Primary outcomes were conditional associations between nodes (edge weights), centrality of nodes (betweenness, closeness and strength) and clusters (groups of co-occurring nodes). The secondary measures were p-values from the Network Comparison Test.

**Results:**

The strongest conditional association was between depressive and anxiety symptoms (edge weight=1.95 for adolescents and 1.72 for adults). Family history of mental illness was associated with spiritual help-seeking among adult women (edge weight=0.19) and adolescent girls (edge weight=0.17) and with professional help-seeking among adult men (edge weight=0.05). Adverse experiences and mental health symptoms were not associated with any form of help-seeking among adolescent boys. Depressive symptoms had the highest strength (3.22) in the adolescent network, whereas spiritual help-seeking was the most central node in the adult network (betweenness=15, closeness=0.03 and strength=3.21). Four small clusters were identified in the adolescent network, with violence victimisation and substance use behaviour present in all clusters. Two clusters were identified in the adult network: one comprising depressive and anxiety symptoms, violence victimisation and spiritual help-seeking; and the other comprising family history of mental illness, neighbourhood disorganisation, violence victimisation and spiritual help-seeking.

**Conclusion:**

Our findings may inform strategies to promote help-seeking and improve the responsiveness of mental health support for adolescents and adults.

**STRENGTHS AND LIMITATIONS OF THIS STUDY**

- The study was reported in accordance with the Strengthening Reporting of Observational Studies in Epidemiology (STROBE) checklist for cross-sectional studies.
- Sensitivity and stability analyses supported the robustness of the results, while also indicating associations that require cautious interpretation due to lower stability.
- The cross-sectional design of the study limits causal inference.
- Participants were recruited from schools, potentially limiting the generalisability of the findings to non-school based adolescent and adult population in Ghana.

## Introduction

 Adverse experiences including neighbourhood deprivation and violence victimisation are social determinants of mental health that are highly prevalent, affecting the lives of millions of adolescents and hundreds of adults worldwide.^1 2^ Adverse experiences seldom occur in isolation; they cluster and interact in ways that contribute to the onset and persistence of mental health symptoms.^3^ Emerging evidence suggests mental health symptoms may increase vulnerability to subsequent adverse experiences.^4^ Adverse experiences and mental health symptoms, if unaddressed, may have consequences extending beyond affected adolescents and adults to families, educational institutions and health systems through caregiving demands, educational disruption and lost productivity.^5^

### Associations between adverse experiences and mental health

Although most adolescents and adults live in low- and middle-income countries (LMICs) including Ghana, research on associations between adverse experiences and mental health symptoms has largely originated from high-income countries.^5^ Studies from Ghana suggest the association between adverse experiences and mental health symptoms varies according to the type of adverse experience, mental health symptom, and gender being examined.^6 7^ For example, violence victimisation has been associated with depressive and anxiety symptoms, whereas accident involvement and neighbourhood disorganisation have shown gender-specific associations with anxiety symptoms among young people in Ghana.^7^ However, previous studies have often examined adverse experiences either as individual predictors or correlates of mental health symptoms, or as exerting equal and independent effects on mental health symptoms.^8 9^ These approaches, eg, single-event approach and cumulative scores approach, may overlook the apparent effects of co-occurring adverse experiences, and may be biased towards the implicit assumption that the effect of one adverse experience will not vary in relation to the presence of other adverse experiences.^10^ These approaches may not fully capture the complex association between adverse experiences and mental health symptoms.^11^

Network analysis can be used to examine the complex associations between adverse experiences and mental health symptoms, while accounting for their co-occurrence.^11^ Network analysis can also be used to estimate the strength and structure of the conditional associations between adverse experiences and mental health symptoms.^11 12^ In network models, adverse experiences and mental health symptoms represent nodes, while edges represent associations between pairs of nodes conditional on the other nodes in the network.^11 12^ Previous studies have used network analysis to examine the association between adverse experiences and emotional and behavioural problems in adolescence, as well as associations between adversities experienced during childhood and adolescence and mental health symptoms in adulthood.^3 13 14^ One of these studies found that adverse experiences such as abuse and bullying victimisation were associated with emotional or behavioural problems, whereas financial difficulties and parental unemployment were more strongly associated with other adverse experiences than with emotional or behavioural problems, after accounting for other nodes in the network.^3^

However, most previous network studies have focused on adverse childhood experiences, including maltreatment and family-related adversity, and their association with emotional or behavioural problems, and mental health symptoms in childhood, adolescence or adulthood.^3 13 14^ Less attention has been given to adverse experiences occurring during adolescence and adulthood, despite evidence suggesting the types of adverse experiences relevant to mental health may differ across the life course.^15^ In Ghana, adverse experiences including accident involvement, neighbourhood disorganisation and incohesion, school-related stress and violence victimisation are prevalent among adolescents and adults.^6^ Our study extends existing literature by examining the conditional associations between adverse experiences and mental health symptoms (ie, depressive and anxiety symptoms) in separate networks for adolescents and adults in Ghana. Comparing these networks may help determine whether the two age groups differ in network structure (ie, the pattern and strength of conditional association between adverse experiences and mental health symptoms) and global strength (ie, the strength of all associations in the networks).^11 16^ Previous network studies have also used centrality measures (ie, betweenness, closeness and strength) to examine the relative importance of nodes within a network.^11 13 17^ Centrality measures were examined in this study to identify adverse experience and mental health symptoms warranting further consideration as potential targets for intervention.^11^

### Clustering of adverse experiences and mental health problems

Beyond conditional associations, network analysis can be used to examine the clustering of adverse experiences and mental health symptoms among adolescents and adults.^11^ Theoretical models including the dimensional model of adversity and psychopathology and the harshness-unpredictability framework suggest that some adverse experiences may share similar underlying characteristics, such as threat, deprivation, harshness and unpredictability.^9 18^ Previous network studies have identified clusters of adverse experiences broadly consistent with these theoretical models.^9 19^ A more recent network study examining the clustering of adverse experiences and emotional and behavioural problems among children found that bullying victimisation, peer rejection and emotional problems formed a cluster (ie, groups of highly interconnected nodes), whereas other adverse experiences such as financial difficulties, parental addiction, parental divorce, physical and sexual abuse formed separate clusters that did not include emotional or behavioural problems.^3^ These findings suggest that some adverse experiences may cluster with emotional or behavioural problems, whereas others may cluster mainly with other adverse experiences.^3^ To our knowledge, no previous network study has examined the clustering of adverse experiences and mental health symptoms among adolescents and adults in Ghana. Examining the clustering of adverse experiences and mental health symptoms among adolescents and adults in Ghana may help identify which adverse experiences cluster together, whether depressive and anxiety symptoms cluster with particular adverse experiences and whether these clusters differ between age groups.^11^ Identifying clusters would also inform the development of preventive strategies and mental health support tailored to the needs of adolescents and adults experiencing adversities and mental health symptoms in Ghana.^20^

### Extending adversity–mental health networks to include help-seeking

To our knowledge, no studies based on network analysis have yet included help-seeking, an important behaviour whose absence or presence can increase or buffer the effect of adverse experiences and mental health symptoms among adolescents and adults, particularly in Ghana where both adverse experiences and mental health symptoms are notably high.^21 22^ Help-seeking refers to the actions through which individuals recognise adverse experiences and mental health symptoms, interpret its cause and pursue support from available and accessible sources within their sociocultural context.^23^ In Ghana and other LMICs, help-seeking often involves movement across different systems of care.^24^ Professional help-seeking involves seeking support from mental health professionals including psychiatrists, psychologists, mental health nurses and general practitioners.^25^ Spiritual help-seeking involves seeking support from traditional and faith-based healers including pastors, imams, prophets, prayer camps, herbalists or diviners who are often perceived as addressing the spiritual or moral dimensions of adverse experiences and mental health symptoms.^24 25^ A network including adverse experiences, mental health symptoms and help-seeking may help identify whether adolescents and adults experiencing adversities and mental health symptoms seek help at all and, if so, whether the same adverse experiences and mental health symptoms are associated with both forms of help-seeking or whether professional and spiritual help-seeking are associated with different adverse experiences and mental health symptoms.

Gender may be relevant when examining associations between adverse experiences, mental health symptoms and help-seeking among adolescents and adults in Ghana. Gender-based socialisation and parenting practices in Ghana place different expectations on boys and girls.^6^ Boys, for example, may be socialised to be strong, hardy and able to withstand adversity, whereas girls may be socialised differently and perceived as requiring greater protection.^26^ Gender-based socialisation may influence exposure and disclosure of adverse experiences and mental health symptoms.^6^ Help-seeking attitudes and behaviours are also influenced by gender norms and expectations.^25^ For example, masculine norms emphasising emotional restraint, strength and self-reliance may discourage help-seeking among adolescents and adults experiencing adversities and mental health symptoms.^25^ Therefore, examining gender differences and similarities may inform the development of trauma-informed interventions and preventive strategies tailored to the gender-specific needs of adolescents and adults in Ghana. This study aimed to examine the (1) conditional associations between adverse experiences, mental health symptoms and help-seeking, represented as nodes within the adolescent and adult networks, including gender-specific associations, (2) centrality of nodes within the adolescent and adult networks and (3) clustering of adverse experiences, mental health symptoms and help-seeking among adolescents and adults in Ghana.

## Methods

This study is reported in accordance with the strengthening the reporting of observational studies in epidemiology (STROBE) checklist for cross-sectional studies (see online supplemental table 1).^27^

### Study design and setting

This cross-sectional study was conducted in three indigenous senior high schools located in the Central, Eastern and Greater Accra regions of Ghana.^6^ A school was considered indigenous if over 90% of the student population is from the region or district in which the school is located.^28^ In Ghana, senior high schools primarily enrol adolescents aged 12–17 years.^29^ However, adults who did not complete secondary education earlier in life may re-enter senior high school to pursue formal education through the free senior high school policy and national commitments to adult education and lifelong learning.^30^ Consequently, study participants consisted predominantly of adolescents and a small number of adults.

### Participants

Participants are students enroled in the three indigenous senior high schools and present on the day of data collection.^6^ Eligibility required enrolment in the selected schools.^6^ Students with a documented diagnoses of mental disorders were excluded from the study to minimise potential confounding due to severity of existing mental disorders and treatment, when assessing mental health symptoms. No students with sensory impairment (e.g., visual impairments) were identified at any of the three schools.^31^ The sample size was determined by the number of students enroled in the selected schools and present on the day of data collection. A total of 1886 participants were eligible to participate in this study; however, two participants were excluded due to missing demographic information (eg, age and gender) required for the analysis.^6^

### Sampling and recruitment procedure

A multistage sampling technique was used to select the schools.^6^ Districts (ie, small administrative units) were randomly selected within each region.^6^ Within each selected district, one indigenous senior high school was purposively selected, see figure 1 for the flow chart of sample selection.^28^ Officials from the District Education Office of the various districts in the three regions provided information on schools meeting the indigenous Senior High School criteria. They also granted institutional permission for the study.^6^

**Figure 1:**
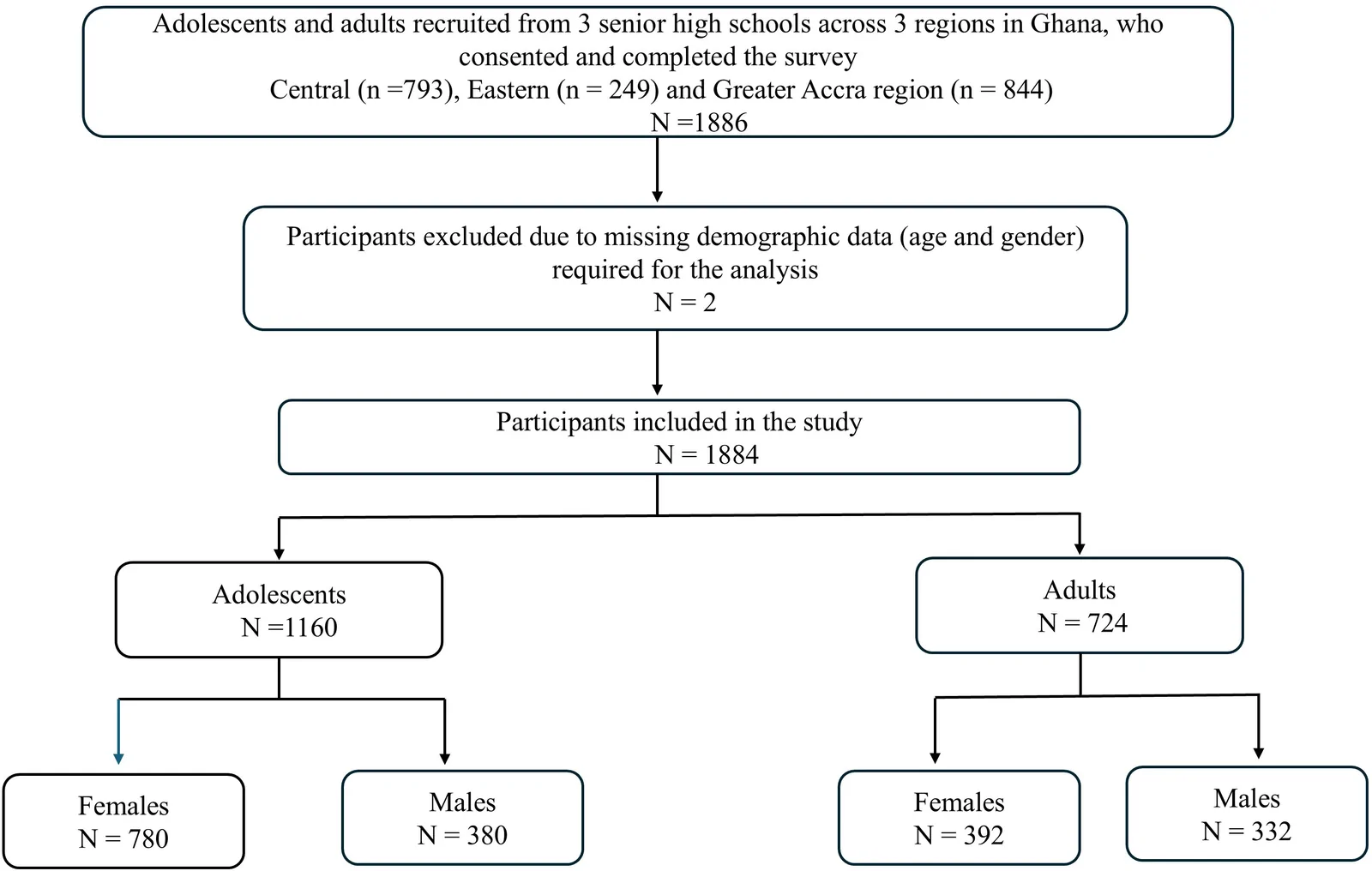
Flow chart of sample selection.

### Measures

Adverse experiences were assessed using self-reported measures across seven domains encompassing personal, family and neighbourhood-level events.^6^ These included accident involvement, family history of mental illness, living in disorganised neighbourhood, living in incohesive neighbourhood, physical violence victimisation (hereafter violence victimisation), school stress and substance use behaviour.^6^ Substance use behaviour is more commonly classified as an adverse health behaviour; however, in this study, we conceptualised it as an adverse experience in recognition of the significant health, social and psychological consequences it carries in the Ghanaian context.^32^ Importantly, these adverse experiences have been considered in previous studies.^6 33^ Responses were categorical (Yes/No). Details of the measures of adverse experiences are provided in online supplemental table 2.

Mental health symptoms. The Patient Health Questionnaire 9-item version (PHQ-9) was used to assess depressive symptoms.^34^ The Generalised Anxiety Disorder 7-item scale (GAD-7) was used to assess anxiety symptoms.^35^ Both screening measures assess symptom frequency over the preceding 2 weeks using a 4-point Likert scale ranging from 0 (not at all) to 3 (nearly every day). Total scores range from 0 to 21 for the GAD-7 and from 0 to 27 for the PHQ-9.^34 35^ Scores were retained in their continuous format and dichotomised using a standardised cut-off (≥10) to indicate the presence or absence of depressive symptoms or anxiety symptoms.

Help-seeking. Professional help-seeking was assessed by asking participants whether they had sought mental health support from a mental health professional (eg, psychologist, psychiatrist and nurse), whereas spiritual help-seeking was assessed by asking whether participants had sought support from traditional and faith-based healers (eg, herbalist, pastor and traditional healer (Odusini)).^6^ Responses were categorical (Yes/No).

Socio-demographic characteristics were self-reported and included age (in years), gender (man/woman; boy or girl), area of residence (rural, urban or peri-urban) and physical activity status (number of days in the past month on which participants engaged in at least ten minutes of vigorous physical activity such as running, cycling, among others).^6^

### Questionnaire pre-testing

Measures for mental health symptoms, namely the GAD-7 and PHQ-9, have been validated for use in the Ghanaian context.^6^ In this study, the Cronbach’s alpha coefficient of the GAD-7 and PHQ-9 was 0.78 and 0.72, respectively.^6^ The adverse experiences and help-seeking measures have not been validated in the study population. Therefore, these measures were pre-tested to ensure questions work as intended and are understood by individuals similar to the study population.^36^ Questionnaire pre-testing was conducted in two stages: First, ten students recruited from Achimota Senior High School in the Greater Accra region of Ghana reviewed the adverse experiences and help-seeking questions for clarity and comprehensibility. Their feedback indicated that the questions were appropriately worded and understandable; therefore, no changes were made. Thereafter, the complete study questionnaire was pre-tested among 25 students in the same school to identify any issues with the questionnaire and data collection procedure, as well as to estimate the completion time. No issues were identified during this stage.

### Data collection

Data were collected in June–September 2019 using a structured questionnaire administered in a group test format. The research team (ie, two males and two females with degree qualification in Nursing or Psychology) explained the purpose of the study and administered the questionnaire to only those who consented to participate in the study.^6^ Participants were informed that there were no right or wrong answers and encouraged not to copy from peers. Participants completed the questionnaire individually while seated together in their regular classroom setting. The research team supervised data collection across all participating schools.

Ethical approval was obtained from the Noguchi Memorial Institute for Medical Research Institutional Review Board of the University of Ghana (NMIMR-IRB CPN 012/19–20).^6^ Study procedures are in accordance with the Declaration of Helsinki.^37^ Prior to fieldwork, the research team received training on participant recruitment, questionnaire administration, data quality assurance and ethical principles including confidentiality and informed consent. Written and verbal informed consent was obtained from all adult participants. Adolescents aged 12–17 years provided assent, while informed consent was obtained from a parent or legal guardian. Participation was voluntary, and ethical principles relating to confidentiality and the right to withdraw from the study were clearly communicated and upheld.^6 37^ A clinical psychologist was available to provide remote psychological support if needed, and the research team collaborated with school counselling units to ensure follow-up support for any participant requiring additional assistance.

### Patient and public involvement

Patient and public contributors were not involved in the design, conduct, reporting or dissemination planning of this study.

### Analysis

Analyses were conducted in R (V.4.3.3) within the RStudio environment (V.2025.09.2+418). Codebase used for the analyses has been registered on the open science framework (OSF) platform https://doi.org/10.17605/OSF.IO/TH5GX.^38^ The published dataset is available at https://data.mendeley.com/datasets/nvmnbnnmzm/1.^39^ All analyses were stratified by age group and organised into two stages: descriptive statistics and network analysis (see figure 2 for schematic overview of the analyses).

**Figure 2:**
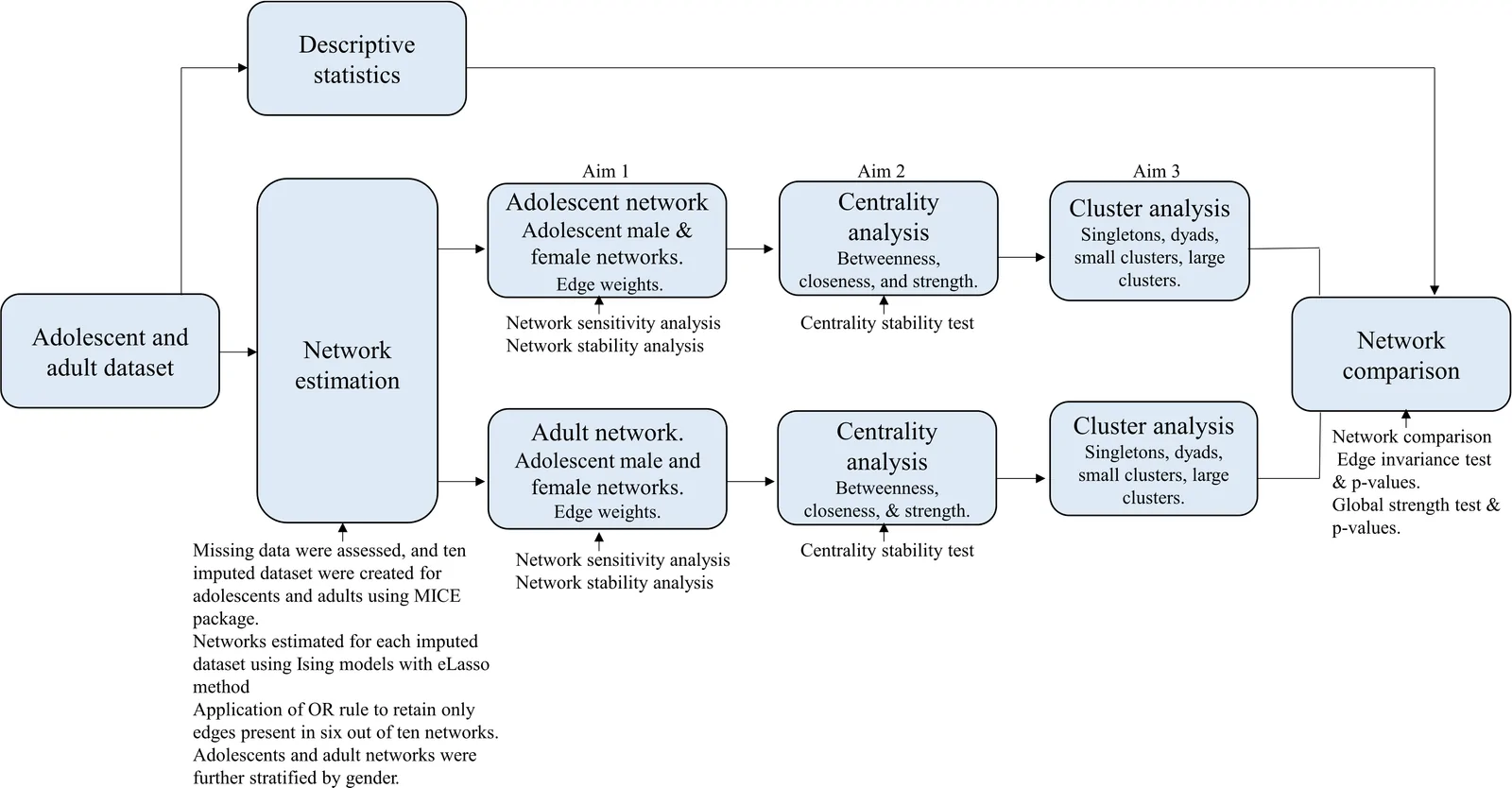
Schematic overview of analyses. MICE, Multiple Imputation by Chained Equations.

### Stage 1. descriptive statistics

Descriptive statistics including mean, standard deviations and percentages were used to summarise socio-demographic characteristics and distribution of adverse experiences, mental health symptoms and help-seeking among adolescents and adults.

### Stage 2. network analysis

Network analysis was then conducted in four steps: network estimation and visualisation, centrality analysis, cluster analysis and network comparison.

#### Network estimation and visualisation

Data were examined at the variable level to identify the extent and pattern of missingness prior to network estimation. Family history of mental illness was found to be the node with the most missing data (n=615, 32.6%); see online supplemental table 3 for multiple imputation report, and online supplemental table 4 for listwise data loss report. Logistic regression results supported the plausibility that data was missing at random, see supplementary R codebase in analysis code registered on, see supplementary R-codebase and output in analysis code registered on the OSF platform https://doi.org/10.17605/OSF.IO/TH5GX.^38^ Multiple imputation by chained equations package (V.4.3.3) was used to generate ten imputed datasets for adolescents and adults, respectively. Multiple imputation included measures of adverse experiences, mental health symptoms and help-seeking; but no additional predictive variables, eg, age, gender, physical activity status. The Ising model with the least absolute shrinkage and selection operator was then applied to each imputed dataset to estimate networks separately for adolescents and adults. The Ising model was chosen because it is a commonly used model designed to construct networks from binary data.^11 40 41^ The Extended Bayesian Information Criterion with a hyperparameter y=0.25 was used to balance fit and the sparsity in the adolescent and adult networks.^12^ Edges were selected using the OR rule (ie, an edge is included in the networks when either node involved in that edge (eg, depressive symptoms and violence victimisation) predicts each other, and only edges present in at least 6 of the 10 imputed networks were retained in the final adolescent and adult networks. A similar strategy has been used in previous studies.^3 42^ Adolescent and adult networks were also stratified by gender to explore potential subgroup variations.

Sensitivity analyses were conducted where adolescent and adult networks were re-estimated using: (i) the AND rule with hyperparameter y=0.25, which retains edges only when both nodes predict each other,^43^ (ii) the OR rule with hyperparameter y=0.00; (iii) a randomly selected subsample of 724 adolescents to match the adult sample size, and (iv) a listwise deletion approach to explore the robustness of the imputation procedure, to ensure that reported conditional associations were not spurious results arising from methodological choices. Non-parametric bootstrapping (1000 bootstraps) with 95% CIs was performed to assess the stability of the edge weights and to determine associations that were stable and those that should be interpreted with care.^12^

Adolescent and adult networks were visualised using the qgraph package (V.1.6.9), and the Fruchterman-Reingold algorithm was used to determine the layout of the networks.^11^ The outputs of this step included networks for adolescents and adults, adolescent and adult networks stratified by gender, re-estimated networks and bootstrapped stability plots. In the adolescent and adult networks, nodes represented adverse experience, mental health symptoms or help-seeking, and the size of each node was proportional to its prevalence, so that more frequently endorsed nodes appeared larger in the networks. Importantly, adolescent and adult networks were undirected and edges represented conditional associations between the nodes rather than causal associations.^3 11^ Conditional association refers to the association between two nodes after controlling for all other nodes in the network.^3^ Edge weights (ie, strength of conditional association between two nodes) were used for network inference. We categorised associations as strong (edge weight, r>0.50), moderate (r=0.30–0.50) or weak (r<0.30) conditional associations.^44^

#### Network centrality analysis

Centrality indices including betweenness (ie, the number of times a specific node lies between two other nodes on their shortest connecting path), closeness (ie, the inverse of the average length of all shortest paths between a node and all other nodes) and node strength (ie, the sum of all edge weights incident to a given node) were examined to assess the relative importance of nodes in the adolescent and adult networks.^11 17^ The stability of centrality indices was assessed using the case-dropping bootstraps, with a centrality stability coefficient (CS-coefficient) above 0.50 considered stable.^12 14^ The outputs of the centrality analyses included the betweenness, closeness and strength values for each node visualised in bar plots, CS-coefficient and stability plots.

#### Network cluster analysis

Cluster analysis was conducted through visual inspection of the adolescent and adult networks. This approach was chosen because it is appropriate for smaller networks.^45^ Our approach is also consistent with existing literature suggesting that computational clustering methods (eg, louvain algorithm and walkrap algorithm) should be applied with caution in very small networks, because the assumptions underpinning these methods may disproportionately influence cluster assignments, particularly for weaker associations.^44^ Outputs of the cluster analysis included groups of nodes categorised as singletons (ie, one node with no incident edges), dyads (ie, pairs of adjacent nodes with other incident edges) and small (3–5 nodes) and larger clusters (> 5 nodes).

#### Network comparison

The Network Comparison Test (NCT) with 1000 permutations was used to examine global strength invariance (ie, whether the total strength of all associations in the network, expressed as the sum of edge weights, is similar between the adolescent and adult networks) and edge invariance (ie, whether the strength of specific associations between nodes in the networks is similar across the two groups). The main output for this analysis included p-values, where p<0.05 indicated a significant difference between the adolescent and adult networks.^16^

## Results

### Descriptive statistics

A total of 1884 participants were included in this study. About 61.6% of the participants were adolescents aged 12–17 years. Most adolescents were females (n=780, 67.2%), grew up in urban areas (n=548, 47.9%) and were not physically active (n=475, 60.4%). About 38.4% of the participants were adults aged 18–45 years. Most adults were females (n=392, 54.1%), grew up in rural areas (n=262, 36.8%) and were not physically active (n=323, 65.8%). For both adolescents and adults, living in disorganised neighbourhood was the most prevalent adverse experience (adolescents, n=753; adults, n=490), depressive symptoms was the most prevalent mental health symptoms (adolescents, n=356; adults, n=232), and professional help-seeking was the most prevalent form of help-seeking (adolescents, n=213; adults, n=185) (see online supplemental table 5 for the descriptive statistics).

### Associations between adverse experiences, mental health symptoms and help-seeking among adolescents and adults (Aim 1)

Figure 3 presents the network structure of conditional association between adverse experience, mental health symptoms and help-seeking among adolescents and adults. The adolescent network comprised 15 edges, while the adult network comprised 12 edges. In both adolescent and adult networks, we found a strong and stable association between AE3 ‘living in disorganised neighbourhood’ and AE4 ‘living in incohesive neighbourhood’ (r=1.53, adolescents; r=1.63, adults), MH1 ‘depressive symptoms’ and MH2 ‘anxiety symptoms’ (r =1.95, adolescents; r=1.72, adults) and HS1 ‘professional help-seeking’ and HS2 ‘spiritual help-seeking’ (r=3.05, adolescents; r=2.69 adults); see online supplemental figure 1 and 2 for non-parametric bootstrapped plots; and online supplemental tables 6 and 7 for additional associations identified in the networks.

**Figure 3:**
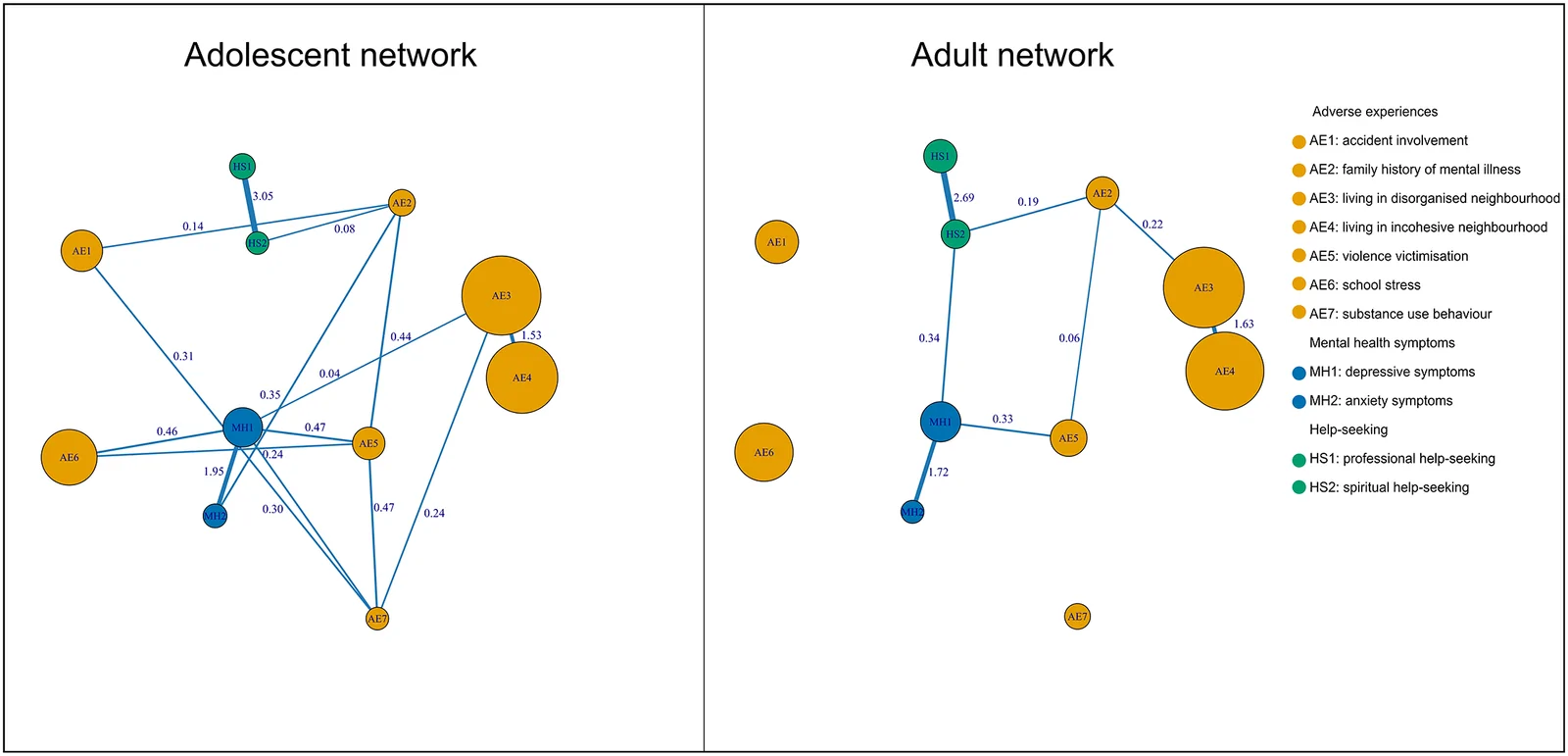
Network structure of associations between adverse experiences, mental health symptoms and help-seeking among adolescents and adults. Blue edges represent positive association. A thicker edge represents stronger associations between the nodes, while thin edges reflect weak associations. The number on the edges represents edge weights, which is the strength of the association.

Sensitivity analysis showed that most of the conditional associations between adverse experiences, mental health symptoms and help-seeking were robust to methodological choices. First, most associations identified in the adolescent and adult networks were also retained in the networks re-estimated using the AND rule. Only three edges in the adolescent network (ie, depressive symptoms and living in disorganised neighbourhood, accident involvement and family history of mental illness and family history of mental illness and spiritual help-seeking), and adult network (ie, violence victimisation and family history of mental illness, family history of mental illness and living in disorganised neighbourhood and family history of mental illness and spiritual help-seeking) failed to meet the stricter requirement of reciprocal prediction of the AND rule, and were not retained in re-estimated networks. Second, re-estimating the adolescent and adult networks using the OR rule with a hyperparameter y=0.00 led to the inclusion of four additional edges (ie, accident involvement and violence victimisation, violence victimisation and living in disorganised neighbourhood, family history of mental illness and living in disorganised neighbourhood and family history of mental illness and living in incohesive neighbourhood) in the adolescent network, but the re-estimated adult network was identical to the main adult network. Third, adolescent network re-estimated using a random subsample of 724 adolescents did not differ from the main adolescent network. Fourth, using listwise deletion to handle missingness led to the exclusion of six edges from the adolescent network (ie, anxiety and family history of mental illness, accident involvement and family history of mental illness, accident involvement and substance use behaviour, violence victimisation and professional help-seeking, substance use behaviour and professional help-seeking, family history of mental illness and spiritual help-seeking), and two edges from the adult network (ie, depressive symptoms and spiritual help-seeking, and family history of mental and spiritual help-seeking); see online supplemental figures 3-8 for results on sensitivity analyses. Overall, associations that appeared or disappeared in the re-estimated networks were primarily weak or moderate, showed instability across the bootstrapped resampling procedure, and hence should be interpreted cautiously.

### Associations between adverse experiences, mental health symptoms and help-seeking among adolescents and adults by gender

Figure 4 shows the adolescent network stratified by gender. In the adolescent boys’ network, AE2 ‘family history of mental illness’ was associated with both AE4 ‘living in incohesive neighbourhood’ (r=0.02) and MH2 ‘anxiety symptoms’ (r=0.15). AE3 ‘living in disorganised neighbourhood’ was associated with MH2 ‘anxiety symptoms’ (r=0.03). In the adolescent girls’ network, AE2 ‘family history of mental illness’ was associated with AE1 ‘accident involvement’ (r=0.03), AE5 ‘violence victimisation’ (r=0.35), MH2 ‘anxiety symptoms’ (r=0.14) and HS2 ‘spiritual help-seeking’ (r=0.17). MH1 ‘depressive symptoms’ was associated with both AE5 ‘violence victimisation’ (r=0.38) and AE6 ‘school stress’ (r=0.36).

**Figure 4:**
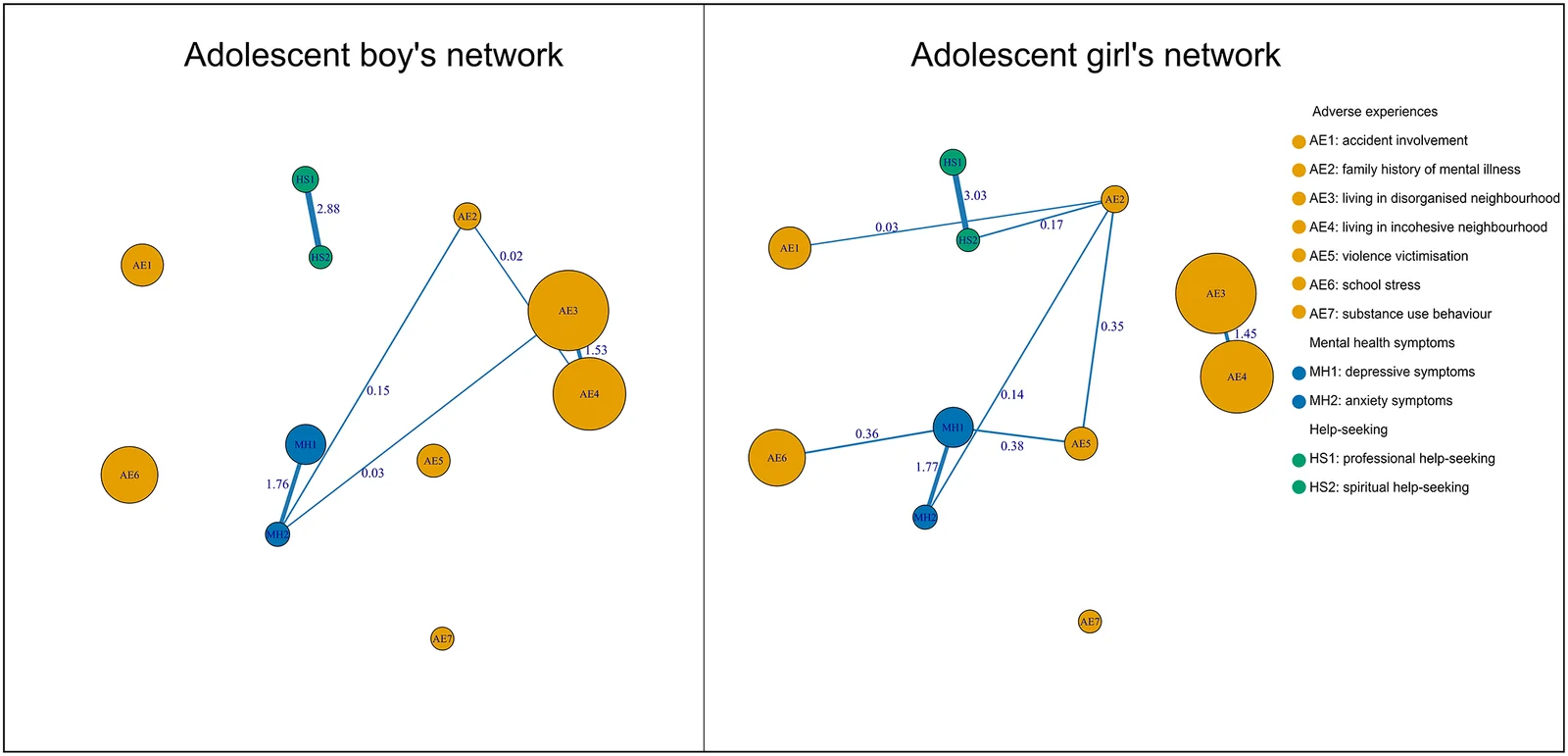
Adolescent network structure of adverse experiences, mental health symptoms and help-seeking stratified by gender.

Figure 5 shows the adult network stratified by gender. In the men’s network, AE2 ‘family history of mental illness’ was associated with AE3 ‘living in disorganised neighbourhood’, AE6 ‘school stress’, AE7 ‘substance use behaviour’ and HS1 ‘professional help-seeking’.

**Figure 5:**
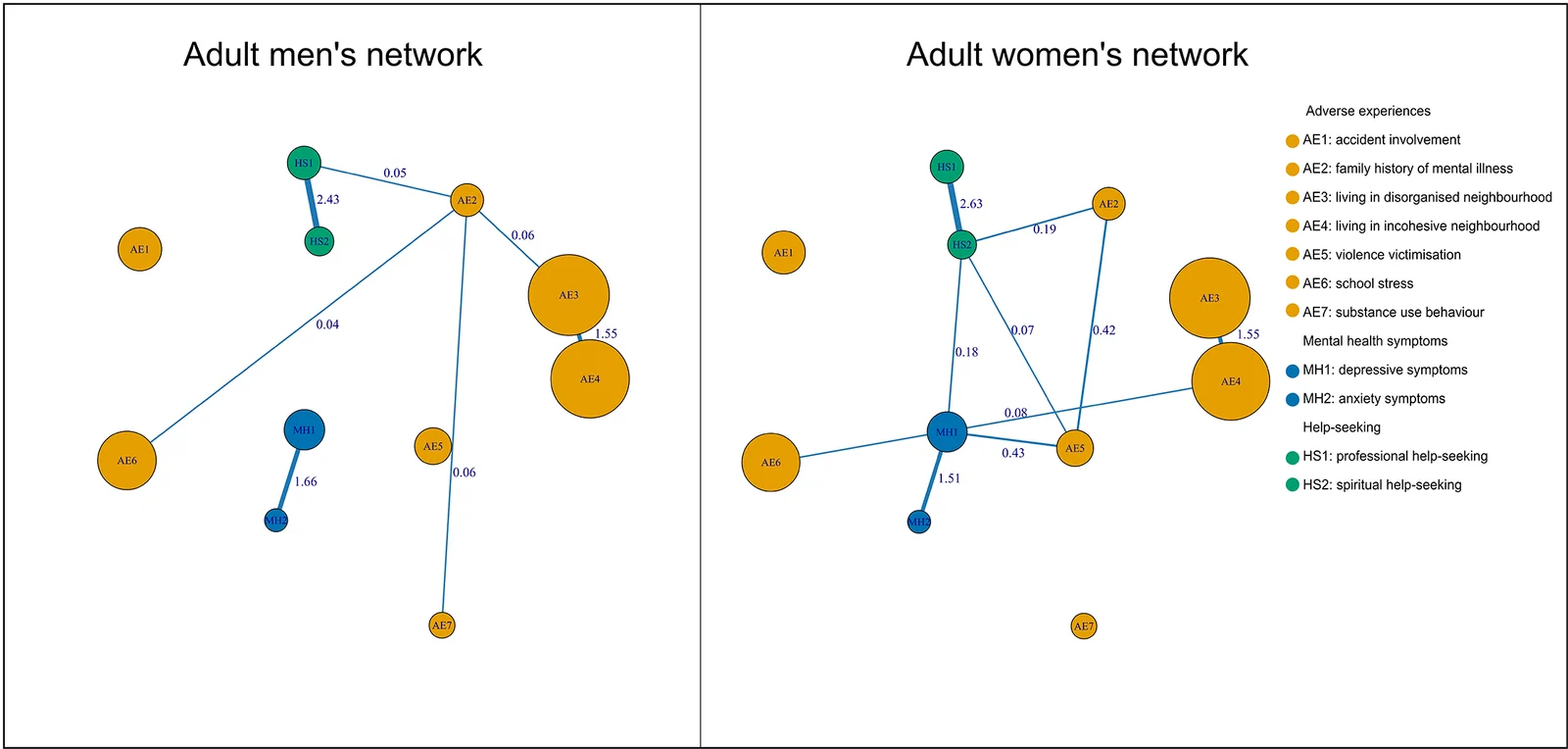
Adult network structure of adverse experiences, mental health symptoms and help-seeking stratified by gender.

In the women’s network, AE2 ‘family history of mental illness’ was associated with AE5 ‘violence victimisation’ and HS2 ‘spiritual help-seeking’. AE5 ‘violence victimisation’ was associated with HS2 ‘spiritual help-seeking’. MH1 ‘depressive symptoms’ was associated with AE5 ‘violence victimisation’ and HS2 ‘spiritual help-seeking’. AE4 ‘living in an incohesive neighbourhood’ was associated with AE6 ‘school stress’.

### Centrality of adverse experiences, mental health symptoms and help-seeking (Aim 2)

In the adolescent network, both AE2 ‘family history of mental illness’ and AE7 ‘substance use behaviour’ had the highest betweenness (16). AE5 ‘violence victimisation’ had the highest closeness (0.02), and MH1 ‘depressive symptoms’ had the highest strength (3.22). In the adult network, HS2 ‘spiritual help-seeking’ had the highest betweenness (15), closeness (0.03); and strength (3.21); see figure 6 for the strength centrality plot, and online supplemental figures 9 and 10 for the betweenness and closeness centrality plots. The CS-coefficient for betweenness and closeness centrality was zero. The strength centrality was stable (> 0.50) for the adolescent network (CS=0.59) and adult network (CS=0.75); see online supplemental figure 11 for centrality stability plots.

**Figure 6:**
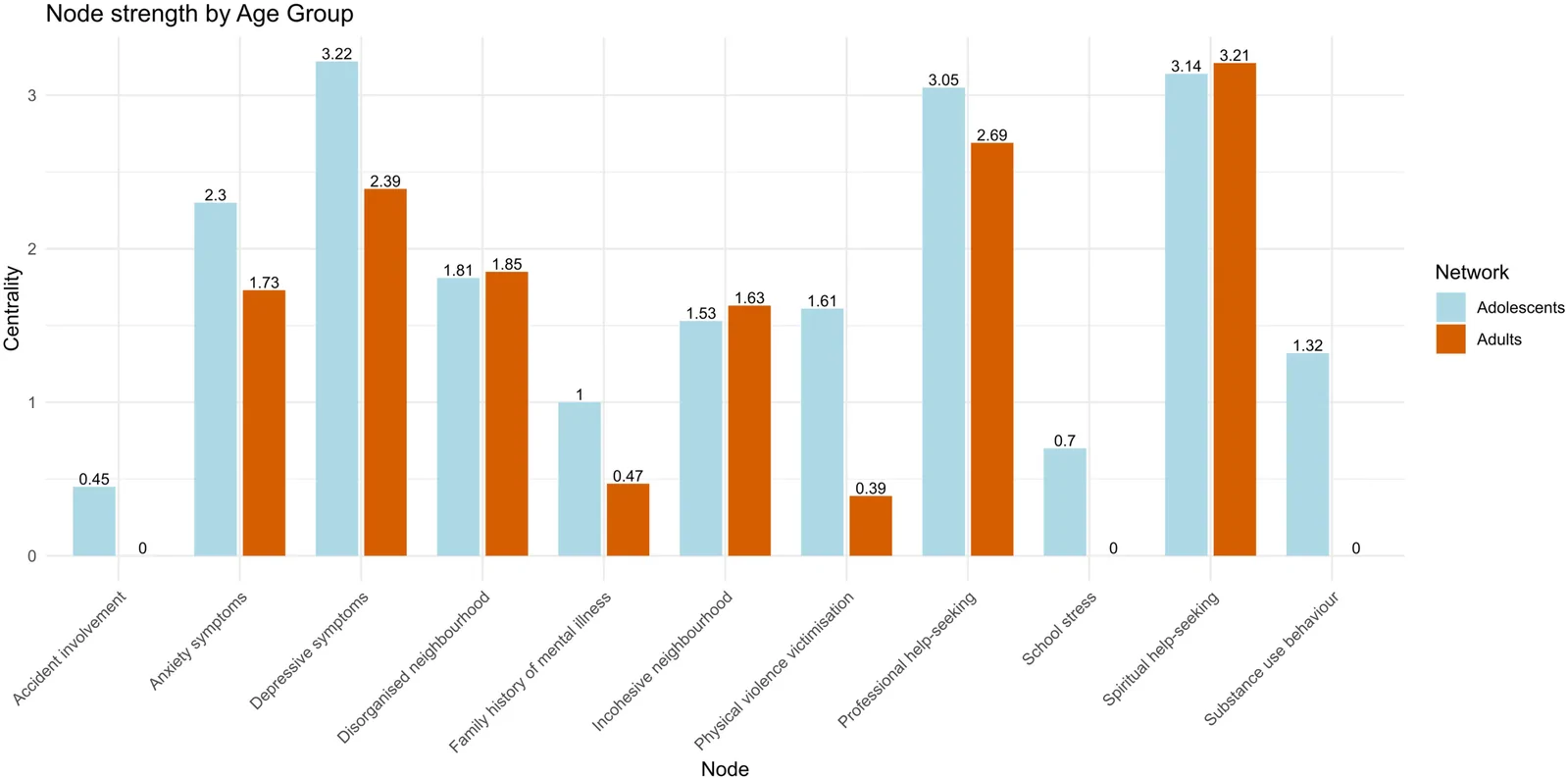
Bar plot illustrates the strength value of each node in the adolescent and adult networks. Higher values indicate higher strength, while lower values represents lower strength.

### Clustering of adverse experiences, mental health symptoms and help-seeking (Aim 3)

In addition to the three strongly associated dyads, we observed four small but weak clusters in the adolescent network. Cluster 1 showed co-occurrence of MH1 ‘depressive symptoms’ with AE3 ‘living in disorganised neighbourhood’, AE5 ‘violence victimisation’, AE6 ‘school stress’, AE7 ‘substance use behaviour’ and MH2 ‘anxiety symptoms’. Cluster 2 showed co-occurrence of AE5 ‘violence victimisation’ with AE2 ‘family history of mental illness’, AE6 ‘school stress’, AE7 ‘substance use behaviour’ and MH1 ‘depressive symptoms’. Cluster 3 showed the co-occurrence of AE7 ‘substance use behaviour’ with AE1 ‘accident involvement’, AE3 ‘living in disorganised neighbourhood’, AE5 ‘violence victimisation’ and MH1 ‘depressive symptoms’. Cluster 4 showed co-occurrence of AE2 ‘family history of mental illness’ with AE1 ‘accident involvement’, AE5 ‘violence victimisation’, MH2 ‘anxiety symptoms’ and HS2 ‘spiritual help-seeking’.

In the adult network, we identified two forms of clusters in addition to the three strong dyads. AE1 ‘Accident involvement’, AE6 ‘school stress’ and AE7 ‘substance use behaviour’ were singletons, that is, nodes with no incident edges. We observed two small but weak clusters. Cluster 1 showed co-occurrence of MH1 ‘depressive symptoms’ with AE5 ‘violence victimisation’, MH2 ‘anxiety symptoms’ and HS2 ‘spiritual help-seeking’. Cluster 2 showed co-occurrence of AE2 ‘family history of mental illness’ with AE3 ‘living in disorganised neighbourhood’, AE5 ‘violence victimisation’ and HS2 ‘spiritual help-seeking’.

### Network comparison test

The global strength of the networks did not differ significantly between adolescents and adults (p=0.52). However, the edge invariance test identified several associations (eg, depressive symptoms and substance use behaviour, accident involvement and substance use behaviour, and depressive symptoms and living in a disorganised neighbourhood) that differed significantly between the adolescent and adult networks, consistent with the results on conditional associations (see online supplemental table 8).

## Discussion

To our knowledge, this study is the first to use network analysis to examine the association and clustering of adverse experiences, mental health symptoms and help-seeking among adolescents and adults in Ghana. We have three main findings in relation to our objectives. First, depressive symptoms and anxiety symptoms, living in disorganised neighbourhood and living in incohesive neighbourhood and professional help-seeking and spiritual help-seeking were strongly associated and appeared relatively stable across all networks, though with evident gender differences. Second, depressive symptoms were the most central node in the adolescent network, while spiritual help-seeking was most central in the adult network. Third, four small clusters comprising different adverse experiences and mental health symptoms were identified in the adolescent network. These clusters were characterised by combinations of family history of mental illness, violence victimisation, substance use behaviour, depressive symptoms, and spiritual help-seeking. However, none of these clusters were associated with professional help-seeking. In the adult network, we observed two small but weak clusters, comprising depressive symptoms, anxiety symptoms, violence victimisation and spiritual help-seeking and another comprising family history of mental illness, living in disorganised neighbourhood, violence victimisation and spiritual help-seeking. Three adverse experiences, including accident involvement, school stress and school stress, appeared as singletons in the adult network. Overall, our findings show that adverse experiences, mental health symptoms and help-seeking co-occur differently for adolescents and adults, underscoring the need for tailored mental health interventions.

In both adolescent and adult networks, the strong association between depressive symptoms and anxiety symptoms is consistent with existing literature suggesting these mental health symptoms frequently co-occur and reinforce each other.^46 47^ Professional help-seeking and spiritual help-seeking were also strongly associated, consistent with the pluralistic help-seeking pathways in Ghana and other LMICs, where adolescents and adults may turn to both traditional and faith-based healers, and mental health professionals for support.^25^ Living in a disorganised neighbourhood and an incohesive neighbourhood were strongly associated, consistent with ecological models suggesting that neighbourhoods perceived as disorganised often also have lower levels of social cohesion.^48 49^ These strong dyadic associations remained stable across bootstrapped sampling, indicating that they appeared consistently across repeated resampling of the data and can therefore be considered reliable.

Gender-specific differences in the association of adverse experiences, mental health symptoms and help-seeking were evident across both age groups. A particularly notable finding was the strong association between violence victimisation and depressive symptoms among women and girls, which is consistent with previous research suggesting that interpersonal violence is associated with poorer mental health outcomes for in this population.^50^ Violence victimisation and family history of mental illness were also associated with spiritual help-seeking among women and girls, suggesting that those who experience violence may be more likely to seek support from traditional and faith-based healers. One plausible explanation is that in Ghana and several African countries, violence victimisation may be interpreted through spiritual explanatory models such as ancestral displeasure, divine punishment, demonic influence, spiritual attack and witchcraft.^51^ These interpretations position pastors, prophets, mallams and traditional healers as familiar and trusted sources of support.^51^ Practices including confession, prayer, seeking spiritual direction and performing rituals therefore become accessible ways to seek protection, meaning and support for depressive symptoms following violence victimisation.^52^ Similar explanatory models may also account for the association between family history of mental illness and spiritual help-seeking.^53^ In addition, women and girls commonly serve as caregivers in Ghana and other LMICs, where limited access to formal mental health services increase reliance on informal support.^54 55^ As caregivers, women and girls may seek help from pastors, prophets, or traditional healers for family members experiencing mental health symptoms, or when mental health concerns arise within the family.^54 55^

Among men, family history of mental illness was associated with both professional help-seeking and substance use behaviour, suggesting that family history of mental illness can simultaneously increase men’s openness to formal mental healthcare and heighten their vulnerability to substance use. Prior research suggests that exposure to mental illness within the family may increase mental health literacy, reduce stigma and improve willingness to seek formal care.^56 57^ At the same time, men who grow up in households affected by mental illness may experience chronic stress, caregiving strain or family conflict, all of which may be associated with increased alcohol and substance use as coping strategies.^55 58 59^ In Ghana, sociocultural norms grant men greater freedom to socialise and take alcohol.^6^ This freedom coupled with beliefs about the medicinal value of alcohol and drugs may further encourage substance use among men with a family history of mental illness.^6 60^ The absence of help-seeking associations with adverse experiences and mental health symptoms among boys further reflects broader patterns of limited engagement with mental health support in this group.^6^ This finding aligns with the patriarchal orientation of Ghanaian society, where adolescent boys are socialised to appear strong, self-reliant and emotionally stoic. Such expectations can make adolescent boys more likely to view seeking help as a sign of weakness, increasing their desire to conceal or deal with difficulties themselves.^6^

The centrality results highlighted nodes that may warrant consideration as potential targets for intervention. Among adolescents, depressive symptoms showed relatively higher strength centrality, indicating that they were more strongly associated to a range of adverse experiences and mental health symptoms compared with other nodes in the network. This finding aligns with existing evidence and possibly suggests the importance of screening for depressive symptoms in this age group.^61^ Among adults, spiritual help-seeking emerged as the node with the highest strength centrality. This finding aligns with research suggesting that spiritual help-seeking is a deeply rooted, widespread practice often serving as the first and sometimes only point of care for adults experiencing adversities and mental health symptoms.^25^ Spiritual help-seeking is especially appealing in the Ghanaian context due to stigma, family privacy norms and limited trust in formal support systems.^62 63^ The relevance of other centrality indices, particularly betweenness and closeness, remain debated in mental health research because their interpretation is not straightforward and they have been shown to be unstable in some cross-sectional networks.^12 64^ Nevertheless, these indices may complement the strength centrality by capturing other aspects of a node’s position within the networks.^64^ In the adolescent network, family history of mental illness and substance use behaviour had the highest betweenness, while violence victimisation had the highest closeness. In the adult network, spiritual help-seeking had the highest betweenness and closeness. The high betweenness and closeness of these nodes may help generate hypotheses about the processes underlying the network structure of adverse experiences, mental health symptoms and help-seeking among adolescents and adults.^64^ However, these findings should be interpreted cautiously.

The cluster results show groups of adverse experiences, mental health symptoms and help-seeking that co-occur within the networks. In the adolescent network, four small but weak clusters were identified. These clusters contained different combinations of adverse experiences, mental health symptoms and spiritual help-seeking, suggesting that adolescents who reported one adverse experience or mental health symptom often reported others, although the specific combinations differed across clusters. This finding aligns with research showing that adverse experiences and mental health symptoms tend to co-occur in different and nuanced combinations among adolescents.^3 14 65^ The heterogeneity observed across clusters suggests that mental health interventions focusing solely on mental health symptoms, without considering co-occurring adverse experiences may not adequately address the needs of adolescents in Ghana. Likewise, interventions designed to address only one cluster (eg, cluster 2, comprising violence victimisation, family history of mental illness, school stress, substance use behaviour and depressive symptoms) without considering other clusters (eg, cluster 3, comprising substance use behaviour, accident involvement, living in a disorganised neighbourhood, violence victimisation and depressive symptoms) may not meet the needs of all adolescents. Further, the repeated presence of family history of mental illness, violence victimisation, substance use behaviour and depressive symptoms across clusters suggests these adverse experiences and mental health symptoms represent potential targets for intervention, and should be explored in future research. In contrast, the adult network showed only two small but weak clusters, and nodes such as accident involvement, school stress and substance use behaviour were singletons, suggesting that these adverse experiences may occur independently of other adverse experiences and mental health symptoms among adults, and may not necessarily be associated with help-seeking.^3^ Further, the two adult clusters showed co-occurrence of depressive symptoms with anxiety symptoms, violence victimisation and spiritual help-seeking, and co-occurrence of family history of mental illness with living in a disorganised neighbourhood, violence victimisation and spiritual help-seeking, suggesting that adults may seek support in response to depressive symptoms and family history of mental illness rather than other adverse experiences. Importantly, these clusters were small and weak, indicating that associations between nodes within each cluster were relatively weak in strength and should be interpreted cautiously.

### Strengths and limitations

This study has several strengths. The measures of depressive and anxiety symptoms used in this study have been validated for use in the Ghanaian context.^6^ Further, stability and sensitivity analyses demonstrated that most conditional associations were stable and not spurious results of modelling choices. Another strength of this study is the inclusion of both adolescents and adults, as well as consideration for gender variation in the association between adverse experiences, mental health symptoms and help-seeking, which may be relevant for informing interventions that are tailored to the needs of adolescents and adults, and responsive to gender differences.

Several limitations should be considered when interpreting these findings. First, there is no consensus in the literature on the definition of ‘adverse experiences’.^8 40^ The present study included seven measures of adverse experience, which may be different from those examined in other studies, limiting comparability of findings to existing research.^8^ Second, definitions of help-seeking vary across studies.^23^ The measures used in this study captured only two forms of help-seeking (ie, professional help-seeking and spiritual help-seeking). Our findings do not reflect the full range of ways adolescents and adults may seek help.^66^ Third, the study did not examine whether adolescents and adults who reported help-seeking received mental health support, or explore the type, quality or outcomes of support received. Fourth, the cross-sectional design of this study precludes causal inference, and the use of self-report measures, including retrospective measures, may have introduced recall bias.^6^

Other limitations include the recruitment of participants from schools, which may limit generalisability of findings to adolescents and adults who are not enroled in school.^6^ Also, strength centrality was the most reliable measure, while betweenness and closeness were less stable, consistent with prior evidence that these centrality indices are prone to instability and should be interpreted cautiously.^12^ Another limitation is that data were collected in 2019 before the COVID-19 pandemic.^6^ Therefore, future research should examine whether these associations and clusters hold in more recent cohorts. Future research should explore evolving forms of adverse experiences (eg, online bullying) and help-seeking (eg, online help-seeking) among adolescents and adults.^66^ Further, the discrepancies between listwise deletion and multiple imputation should be interpreted with caution because the assumptions underpinning listwise deletion were not met.^3^ The use of binarised data, while appropriate for estimating Ising models, may have obscured differences in the frequency of adverse experiences or severity of mental health symptoms.^67^

### Implications for clinical practice, policy and research

#### Clinical practice

Existing literature suggest that screening for adverse experiences and mental health symptoms can support early identification of mental health vulnerability, and help mental health professionals tailor care to individual needs.^68^ In this study, the patterns of association between adverse experiences, mental health symptoms, and help-seeking differed between adolescents and adults. These findings highlight the importance of routine screening for adverse experiences in clinical practice, with consideration of the different needs of adolescents and adults in Ghana. Further, the repeated presence of family history of mental illness across clusters suggests the need for routine inquiry into family history of mental illness and coping strategies employed, particularly among men. The association between depressive symptoms and violence victimisation among women and girls highlights the importance of asking about exposure to violence and safety concerns during mental health assessments, consistent with recommendations for trauma-informed and gender-responsive care.^68^ Finally, existing literature suggests that sociocultural beliefs and norms can influence whether individuals seek professional help or rely on informal sources of support.^25^ Mental health professionals should collaborate with traditional and faith-based healers to help connect adolescents and adults who seek informal support with appropriate mental health services.^69^

#### Policies

The findings point to the potential value of investing in neighbourhood level safety initiatives and school-based prevention programmes that normalise conversations about adverse experiences, mental health symptoms and formal help-seeking.^6^ Also, the repeated appearance of family history of mental illness in all networks supports the value of family-centred policies, such as expanding access to psychoeducation and support for families with history of mental illness.^6^

#### Future research

Future studies should consider person-centred approaches, such as idiographic network models and perceived causal problem networks, which may capture between individual variability more effectively.^65^ Future network studies should use comparable measures where possible, as well as validate adverse experiences and help-seeking measures among adolescents and adults in Ghana, when replicating the study.^40^ Longitudinal designs could help determine whether associations and clusters identified in this study remain stable over time, and clarify potential mechanisms underlying these associations and clusters.^15^ Future research should also include predictive variables in multiple imputation to minimise potential bias.

## Conclusion

This study represents an important first step in understanding the association and clustering of adverse experiences, mental health symptoms and help-seeking among adolescents and adults in Ghana. The findings suggest the need for interventions and research considering the strongly associated dyads, the gender differences in associations, and the centrality of depressive symptoms in adolescence and spiritual help-seeking in adulthood. Cluster analysis results suggest co-occurring adverse experiences and mental health symptoms are not necessarily associated with help-seeking, particularly among adolescents boys. These conclusions should be interpreted cautiously given the limitations of the study, but they nonetheless provide a foundation for future research and intervention development or prioritisation, aimed at improving the mental wellbeing of adolescents and adults in Ghana.

## Supplementary material

10.1136/bmjopen-2026-121157 online supplemental file 1

## Data Availability

Data are available in a public, open access repository. All data relevant to the study are included in the article or uploaded as supplementary information.
