# Supplementary material for: Network analysis of association and clustering of adverse experiences, mental health symptoms and help-seeking among adolescents and adults in Ghana
Source: BMJ Open. 2026 Nov 9;16(11):e121157. doi: 10.1136/bmjopen-2026-121157 (PMC13756331; doi:10.1136/bmjopen-2026-121157)
Supplement: Supplementary file 1 [file bmjopen-2026-121157.reviewer_comments.pdf]

PEER REVIEW HISTORY

BMJ Open publishes all reviews undertaken for accepted manuscripts. Reviewers are asked to complete a checklist review form and are provided with free text boxes to elaborate on their assessment. These free text comments are reproduced below.

ARTICLE DETAILS

Title (Provisional)

Network analysis of association and clustering of adverse experiences, mental health symptoms, and help-seeking among adolescents and adults in Ghana

Authors

Awortwe, Victoria; Daivadanam, Meena; Adjorlolo, Samuel; Woodford, Joanne; Olsson, Erik Martin Gustaf; Magnani, Matteo

---

VERSION 1 - REVIEW

**Reviewer**	**1**
**Name**	**De Vries, Tjeerd Rudmer**
**Affiliation**	**Copenhagen Health Complexity Center**
**Date**	**10-Jun-2026**
**COI**	**NA**

Thank you very much for the opportunity to review the manuscript entitled "Network analysis of adverse experiences, mental health symptoms, and help-seeking among adolescents and adults". The manuscript provides a novel perspective on both conditional associations between adverse experience and mental health due to the local context (Ghana) in which the study was conducted, as well as how help-seeking is involved in this "complex mix" of factors. The main strengths of the study as I see it are as follows: 1) the analysis is rigorous and ample attempts have been made to ensure that the findings are not due to methodological decisions, all of which has been described in great detail ; 2) the discussion in particular is very well written and contextualizes the findings well in relation to the currently available literature. That said, I have a couple of comments that I hope the authors will find useful in further improving their manuscript:

Main comments:

The introduction focusses very much on the methods, and not so much on the networks of adversity and MHP. I would consider rewriting the introduction slightly, leaving out the part about single-adversity approaches & cumulative scoring and write a bit more if one would expect the networks of adversity & mhp to be similar/more densely connected/less densely connected in the local context of this

study (Ghana versus the cited studies - you already mention that adversities are relatively high in the studied context). This would more clearly delineate the added value of this study (besides the added link with help-seeking behavior)

In relation to the help seeking paragraph in the introduction: I feel that a clearer rationale is needed for why the authors zoom in on gender differences (some of the information that is currently included in the introduction could be beneficial to already mention here).

I am not so convinced by the centrality analysis conducted: could the authors elaborate on why this was performed and what the metrics say? Full transparency: I am not a big proponent of centrality indices in these types of networks, as it is often not clear what they mean, conceptually speaking. A focus on the broader "system", i.e. the edges, I think is much more interesting in these types of studies

Does the network clustering analysis take into account the stability of the bootstraps? And can the authors elaborate why this manual approach was chosen over more established, data-driven, clustering approaches?

Moreover, it was not entirely clear what the rationale of the clustering approach was for this study specifically. It was also not directly clear why the network comparison test was performed. I think it is important to clearly outline the rationale of each of the analytical steps taken, most of which should preferably flow directly from the introduction.

Minor comments:

Abstract

I would recommend the authors to focus slightly more on help-seeking behavior already in the abstract, given that this is a main contribution of the manuscript.

Methods

Under study size, it would be great to show the final sample size (and also split by adolescents & adults). The authors could also provide a flowchart of the study to allow the reader to assess how the final sample size was arrived upon

Figures

The network figures are a bit difficult to see with the edge weights sometimes overlapping with the actual edges.

Appendices

I think it would be better to store the analytical code on the OSF (or a similar platform) instead of the supplementary materials. This could help declutter the supplements (i.e. reduce it in size).

**Reviewer**	**2**
**Name**	**Dack, Kyle**
**Affiliation**	**University of Bristol, MRC Integrative Epidemiology Unit**
**Date**	**28-Jun-2026**
**COI**	**NA**

Statistical review:

This manuscript adheres to best practices in the statistical methodology and reporting, and the conclusions are reasonable. I have only a few minor comments to strengthen the reporting.

Abstract:

1. Line 28: It could be informative to add slightly more information about the setting - e.g. adolescents identified in three high schools, and the study dates.

Introduction:

2. The introduction states that this is the first application of network analysis in this subject area, so it is likely that readers may be unfamiliar with the method. There is some explanation provided on lines 92-97, but it is mostly regarding previous applications of the method.

To help readers understand what the study will do, it could be helpful to give a simple and brief overview of the method. This could also explain what the nodes represent, because these are mentioned in the aims on line 121.

Methods:

3. Line 131: Could the specific date range be reported?

4. Line 241: The assessment of missing data and use of multiple imputation is reasonable. However, I have two queries:

-Two participants were excluded due to missing demographic data, whereas missing values for other variables were imputed. What was the rationale for excluding these participants rather than imputing the values? I suspect it is because this was required for categorizing participants into analytical datasets, but this could be stated for readers.

-The replicability could be strengthened by reporting more details of the imputation. Looking at the R code, it appears that the number of iterations was 5 (default parameter), and the imputation included network variables but not demographic variables. Please clarify which variables were included and the rationale for any exclusions, as generally imputation includes as many predictive variables as possible to improve the precision of the imputed values.

5. Supplementary table 3. What does " Adol_724" refer to? It could be clearer with a plain text name.

6. Line 266: How many bootstraps were performed?

Results:

7. The results could be strengthened by reporting the key statistics directly in-text, e.g. most prevalent experiences, weights of the strongest edges, so that readers can follow the key findings without referring to supplementary tables/figures. This would also help as readers may not recall what specific criteria "strong/moderate/weak" refer to.

8. Aside from this, the tables, figures, and in-text reporting are all clear and align with STROBE.

Discussion:

9. The discussion is well written and does not overstate the results - no suggestions.

VERSION 1 - AUTHOR RESPONSE

Reviewer 1:

Comments	Response
The introduction focusses very much on the methods, and not so much on the networks of adversity and MHP. I would consider rewriting the introduction slightly, leaving out the part about single-adversity approaches & cumulative scoring and write a bit more if one would expect the networks of adversity & mph to be similar/more densely connected/less densely connected in the local context of this study (Ghana versus the cited studies - you already mention that adversities are relatively high in the studied context). This would more clearly delineate the added value of this study (besides the added link with help-seeking behavior)	Thank you for your comment. We have revised the introduction by removing the detailed discussion of single-adversity approaches and cumulative scoring. We have also expanded the rationale for examining networks of adverse experiences and mental health problems in Ghana, including whether the network may be similar to, more densely connected than, or less densely connected than those reported in previous studies from other contexts. Please see page 4-7, lines 73-197:<br><br>Introduction<br><br>Adverse experiences including neighbourhood deprivation and violence victimisation are social determinants of mental health that are highly prevalent, affecting the lives of millions of adolescents and hundreds of adults worldwide 1,2. Adverse experiences seldom occur in isolation, they cluster and interact in ways that contribute to the onset and persistence of mental health symptoms 3. Emerging evidence further indicates that mental health symptoms may increase vulnerability to subsequent adverse experiences 4. Adverse experiences and mental health symptoms, if unaddressed, may have consequences that extends beyond affected

	adolescents and adults to families, educational institutions, and health systems through educational disruption, employment difficulties, lost productivity, caregiving demands, and healthcare costs 5.<br><br>Associations between adverse experiences and mental health<br><br>Although most adolescents and adults live in low- and middle-income countries (LMICs) including Ghana, research on associations between adverse experiences and mental health symptoms has largely originated from high-income countries 5. Existing literature from Ghana suggest that the association between adverse experiences and mental health symptoms vary according to the type of adverse experience, mental health outcome, and gender 6,7. For example, violence victimisation has been associated with depressive and anxiety symptoms, whereas accident involvement and neighbourhood disorganisation have shown gender-specific associations with anxiety symptoms 7. However, adverse experiences have often been examined either as separate predictors or correlates of mental health outcomes, or as exerting equal and independent effects on mental health outcomes 8,9. These approaches, e.g., single-event approach and cumulative scores approach, may overlook the apparent effects of co-occurring adverse experiences, and may be biased towards the implicit assumption that the effect of one adverse experience will not vary in relation to the presence of other adverse experiences (Lee et al., 2023). Therefore, these approaches may not fully capture the complex association between adverse experiences and mental health symptoms 12.<br><br>Network analysis can be used to examine the complex associations between adverse experiences and mental health symptoms12. Network allows the structure of associations among adverse experiences and mental health symptoms to be estimated, including the overall pattern and strength of conditional associations across the network 12,13. In network models, nodes represent adverse experiences and mental health symptoms, while edges represent associations between pairs of nodes conditional on the remaining nodes 12,13. Network analysis has been used to examine associations among adverse experiences and emotional and behavioural problems in adolescence, as well as associations between adversities experienced during childhood and adolescence and mental ill-health in adulthood 3,14,15. These studies suggest that adverse experiences do not show the same pattern of association with mental health outcomes once other nodes are considered (Breuer et al., 2020; de Vries et al., 2024). Some adverse experiences retain conditional associations with mental health problems, whereas others are more strongly associated with other adverse experiences3.

However, most previous network studies have focused on adverse childhood experiences, particularly maltreatment and family-related adversity, and their associations with mental health outcomes in childhood, adolescence or adulthood 3,14,15. The present study extends this literature by examining adverse experiences occurring during adolescence and adulthood and their associations with depression and anxiety symptoms among adolescents and adults. This distinction is important because the adverse experiences relevant to mental health may differ across the life course 17. In Ghana, for example, neighbourhood disorganisation is prevalent, while school-related stress, victimisation, head trauma, family mental illness, and substance misuse have also been reported among adolescents and adults 6. A relatively less densely connected network may be plausible if only some adverse experiences retain conditional associations with depression and anxiety symptoms after the other adverse experiences are considered 3. Previous evidence also suggests that some broader ecological adverse experiences may be more strongly associated with other adverse experiences, whereas victimisation-related adverse experiences may be more likely to retain associations with mental health problems 3. These possibilities remain speculative in the Ghanaian context and require empirical evidence. Comparing adolescent and adult networks can therefore establish whether differences occur in particular associations or extend to the overall structure and strength of associations within the networks 12.

Previous network studies have also used centrality metrics to examine the relative importance of a node in the context of other nodes (Breuer et al., 2020; Freeman, 1978). Highly central nodes have sometimes been considered influential and potentially relevant for intervention 14. However, the utility of some centrality metrics, particularly betweenness and closeness centrality, has been questioned in psychological networks 13. Centrality measures were therefore examined in this study because they may highlight nodes that warrant further consideration as potential targets for intervention 12.

Clustering of adverse experiences and mental health problems

Beyond individual associations, network analysis can also examine whether adverse experiences and mental health symptoms form clusters, defined as groups of nodes that are more strongly associated with one another than with other nodes in the network 12. Theoretical models suggest that adverse experiences may cluster according to distinctions between threat and deprivation, where threat involves harm or threat of harm and deprivation involves the absence or insufficiency of expected social, cognitive, or material inputs,

and between proximal and distal sources of adversity, referring respectively to experiences arising in the immediate environment and those arising in the broader ecological context 9,19. Previous network studies have identified communities broadly consistent with these distinctions 9,20.

More recent work that included mental health problems alongside adverse experiences found that bullying victimisation, peer rejection, and emotional problems formed a cluster, whereas family-related, abuse-related, and socioeconomic adverse experiences formed separate clusters 3. This suggests that mental health problems may cluster with particular adverse experiences 3. Comparable evidence from Ghana is limited. Examining clusters may therefore help clarify which adverse experiences cluster together, whether depression and anxiety cluster with particular adverse experiences, and whether these clusters differ between adolescents and adults 12. Identifying such clusters may help show which adverse experiences and mental health problems could be considered together in prevention efforts.

Extending adversity–mental health networks to include help-seeking

To our knowledge, no studies based on network analysis have yet included help-seeking, an important behaviour whose absence or presence can increase or buffer the effect of adverse experiences and mental health symptoms among adolescents and adults, particularly in Ghana where both adverse experiences and mental health problems are notably high 21,22. Help-seeking refers to the actions through which individuals recognise adverse experiences and mental health symptoms, interpret its cause, and pursue support from available and accessible sources within their sociocultural context 23.

In Ghana and other LMICs, help-seeking often involves movement across different systems of care 24. Professional help-seeking involves seeking support from biomedical or formal mental health providers such as psychiatrists, psychologists, mental health nurses, and general practitioners 25. Spiritual help-seeking involves seeking support from religious or traditional authorities including pastors, imams, prophets, prayer camps, herbalists, or diviners who are often perceived as addressing the spiritual or moral dimensions of adverse experiences and mental health symptoms 24,25. A network including adverse experiences, mental health symptoms, and help-seeking may help identify whether adolescents and adults experiencing adversities and mental health problems seek help at all and, if so, whether the same adverse experiences and mental health problems are associated with both forms of help-seeking, or whether

	professional and spiritual help-seeking are associated with different adverse experiences and mental health problems.<br><br>When examining associations among adverse experiences, mental health problems, and help-seeking among adolescents and adults, gender may also be relevant. Gender-based socialisation and parenting practices within Ghanaian contexts place different expectations on boys and girls 6. Boys, for example, may be socialised to be strong, hardy, and able to withstand adversity, whereas girls may be socialised differently and perceived as requiring greater protection 26. Such gender-based socialisation may influence exposure to adverse experiences, disclosure of those adverse experiences, and responses to mental health problems6. Help-seeking attitudes and behaviours are also influenced by gender norms and expectations 25. For example, masculine norms emphasising strength, self-reliance, and emotional restraint have been associated with reluctance to seek help for mental health problems 25. Therefore, examining gender differences may inform trauma-informed interventions and prevention efforts that are responsive to gender-specific needs of adolescents and adults in Ghana. This study aims to examine: (1) the conditional association between adverse experiences, mental health symptoms, and help-seeking for adolescents and adults, and across gender; (2) nodes within the adolescent and adult networks that may be relevant for informing intervention priorities; and (3) the clustering of adverse experiences, mental health symptoms, and help-seeking among adolescents and adults in Ghana.
I am not so convinced by the centrality analysis conducted: could the authors elaborate on why this was performed and what the metrics say? Full transparency: I am not a big proponent of centrality indices in these types of networks, as it is often not clear what they mean, conceptually speaking. A focus on the broader "system", i.e. the edges, I think is much more interesting in these types of studies	Thank you for your comment. We fully agree with your concerns regarding the interpretation of centrality indices. This issue was discussed extensively during the preparation of the manuscript, and we considered whether to report these measures at all, focusing instead on edges and clusters. Our rationale for including these measures is as follows:<br><br>Degree/strength centrality identify variables with many and/or strong associations, which may indicate potentially important targets for intervention. Although this information is not unique to a network approach, it is of practical interest. Other centrality measures, while more difficult to interpret in this context, may provide complementary information by capturing aspects of network positions that are not apparent from individual edges alone. For example, high betweenness can arise when a variable is connected to several other variables that are not strongly connected to one another, making it a potentially interesting candidate for further investigation (to answer the question: why is this the case?) or intervention (if the associations indicate influence, as

	suggested by the authors proposing this approach). While we agree that measures such as betweenness do not have a direct mechanistic interpretation in this context, unlike in networks through which entities such as information or pathogens flow, and should therefore be interpreted with caution, unusually high centrality values may nevertheless identify variables occupying distinctive positions in the network. Rather than providing an explanation in themselves, these measures may help generate hypotheses about the processes that gave rise to the observed network structure and may highlight positions that warrant further consideration as potential targets for intervention.<br><br>We have provided brief information about the centrality indices in the introduction. Please see page 5, lines 131-137:<br><br>Previous network studies have also used centrality metrics to examine the relative importance of a node in the context of other nodes (Breuer et al., 2020; Freeman, 1978). Highly central nodes have sometimes been considered influential and potentially relevant for intervention 14. However, the utility of some centrality metrics, particularly betweenness and closeness centrality, has been questioned in psychological networks 13. Centrality measures were therefore examined in this study because they may highlight nodes that warrant further consideration as potential targets for intervention 12.
Does the network clustering analysis take into account the stability of the bootstraps? And can the authors elaborate why this manual approach was chosen over more established, data-driven, clustering approaches?	Thank you for your comments. The network clustering analysis take into account the stability of the bootstraps.<br><br>**Rationale for choosing a manual approach over more established, data-driven, clustering approaches.** Thank you for the comments. In our view, computational clustering is primarily a tool for summarizing large networks. Its main advantages arise when (1) manual inspection is impractical or excessively time-consuming, or (2) cluster structure is already visually apparent and computational methods provide a convenient way to label the resulting groups. When neither of these conditions applies, as is the case with this study, we do not see a clear benefit to using computational clustering over careful manual inspection, which also increases the analyst's engagement with the data and makes analytical decisions more transparent. Furthermore, computational clustering methods should be applied with caution in very small networks. This is particularly important for approaches based on (approximate) null models, such as Louvain

	modularity optimization. In networks with only a small number of nodes and edges, the assumptions underlying these methods may have a disproportionate influence on the resulting clustering. In the present case, the strongest associations would be grouped together regardless of the clustering approach used. By contrast, the weaker associations (which we interpret as potentially interesting signals deserving further investigation, but not as strong enough to support substantive conclusions) may or may not be assigned to the same cluster depending on the specific algorithm or, for stochastic methods, the particular execution. Consequently, computational clustering is unlikely to add substantial information beyond a manual analysis that already recognizes these associations as relatively weak signals and interprets them accordingly.<br><br>We have now provided a rationale for the choice of approach in the manuscript. Please see page 14, lines 389-400:<br><br>Our approach is consistent with existing literature suggesting that computational clustering methods should be applied with caution in very small networks 40. This is particularly important for approaches based on (approximate) null models, such as Louvain modularity optimisation 40. In networks with only a small number of nodes and edges, the assumptions underlying these methods may have a disproportionate influence on the resulting clustering 40. In the present case, the strongest associations would be grouped together regardless of the clustering approach used. By contrast, the weaker associations (which we interpret as potentially interesting signals deserving further investigation, but not as strong enough to support substantive conclusions) may or may not be assigned to the same cluster depending on the specific algorithm or, for stochastic methods, the particular execution 40. Consequently, computational clustering is unlikely to add substantial information beyond a manual analysis that already recognises these associations as relatively weak signals and interprets them accordingly (see Appendix 6 for communities estimated using Walktrap and Louvain algorithms).
Moreover, it was not entirely clear what the rationale of the clustering approach was for this study specifically. It was also not directly clear why the network comparison	Thank you for comments. Network Comparison Test (NCT) with 1000 permutations was used to examine global strength invariance (i.e., whether the total strength of all associations in the network, expressed as the sum of edge weights, is similar between the adolescent and adult networks) and edge

test was performed. I think it is important to clearly outline the rationale of each of the analytical steps taken, most of which should preferably flow directly from the introduction.	invariance (i.e., whether the strength of specific associations between nodes in the networks is similar across the two groups). This information has been provided in the manuscript. Please see page 14, lines 401- 407.<br><br>We have now added additional detail in the introduction to briefly motivate why we perform the network analysis. Please see page 5, lines 128-130:<br><br>Comparing adolescent and adult networks can therefore establish whether differences occur in particular associations or extend to the overall structure and strength of associations within the networks 12.
**Minor comments**	
**Abstract**<br><br>I would recommend the authors to focus slightly more on help-seeking behavior already in the abstract, given that this is a main contribution of the manuscript.	Thank you for your comment. We have now revised the abstract to include help-seeking. Please see page 2, lines 24-30:<br><br>Adverse experiences and mental health symptoms rarely occur in isolation through life; they cluster and contribute to distress across developmental stages. Network analysis is an emerging approach that has been used to examine the complex association between adverse experiences and mental health symptoms. However, no studies based on network analysis have yet included help-seeking, an important behaviour that may facilitate access to support, and mitigate the effects adverse experiences and mental health symptoms among adolescents and adults.
**Methods**<br><br>Under study size, it would be great to show the final sample size (and also split by adolescents & adults). The authors could also provide a flowchart of the study to allow the reader to assess how the final sample size was arrived upon	Thank you for the comment. We have now provided a flow chart of participant selection, please see page 9, lines 233.
**Figures**<br><br>The network figures are a bit difficult to see with the edge weights sometimes overlapping with the actual edges.	Thank you for your comments. We have now ensured that the edge weights donot overlap with the figures. Please see the figures attached.

**Appendices**<br><br>I think it would be better to store the analytical code on the OSF (or a similar platform) instead of the supplementary materials. This could help declutter the supplements (i.e., reduce it in size).	Thank you for your comment. We have now registered the analytical code the OSF platform. Please see page 11, lines 309-311:<br><br>Codebase used for the analyses have been registered on OSF platform https://doi.org/10.17605/OSF.IO/TH5GX

Reviewer 2

Comments	Response
**Abstract**<br>1. Line 28: It could be informative to add slightly more information about the setting - e.g. adolescents identified in three high schools, and the study dates.	Thank you for your comment. We have now added information about the study setting. Please see page 2, lines 35-37:<br><br>We used data from a cross-sectional survey that included 1884 adolescents aged 12-17 years (n =1160) and adult aged 18-48 years (n = 724), recruited from three senior high schools in three regions in Ghana from June-September, 2019.
**Methods**	
Line 131: Could the specific date range be reported?	Thank you for your comment. We have now reported the date range both in the abstract and the main text.<br><br>Please see page 2, lines 35-37: We used data from a cross-sectional survey that included 1884 adolescents aged 12-17 years (n =1160) and adult aged 18-48 years (n = 724), recruited from three senior high schools in three regions in Ghana from June-September, 2019.<br><br>Please see page 10, line 280:<br>Data were collected in June- September 2019 …
Line 241: The assessment of missing data and use of multiple imputation is reasonable. However, I have two queries:<br>-Two participants were excluded due to missing demographic data, whereas missing values for other variables were imputed. What was the rationale for excluding these participants rather than imputing the	Thank you for the comment. Yes, demographic data i.e., age was required for categorising participants into analytical datasets, and that is why we excluded the two participants.<br><br>Please see page 8, lines 221-223:<br>A total of 1,886 participants were eligible to participate in this study; however, two participants were excluded due to missing demographic information (e.g., age and gender), which was required for the analysis

values? I suspect it is because this was required for categorizing participants into analytical datasets, but this could be stated for readers.	
The replicability could be strengthened by reporting more details of the imputation. Looking at the R code, it appears that the number of iterations was 5 (default parameter), and the imputation included network variables but not demographic variables. Please clarify which variables were included and the rationale for any exclusions, as generally imputation includes as many predictive variables as possible to improve the precision of the imputed values.<br><br>Missing data were handled using multiple imputation by chained equations (MICE).	Thank you for your comment.<br><br>We have also provided information about the variables that were included in the multiple imputation.<br><br>Please see page 12, lines 334-336:<br><br>Multiple imputation included measures of adverse experiences, mental health symptoms, and help-seeking; no additional predictive variables, e.g., age, gender, physical activity status<br><br>We did not include age as a predictive variable because it was used to the analytical subgroups and was therefore invariant within each imputation dataset. However, we have acknowledged that not including predictive variables such as physical activity status, place of residence etc. is a limitation because it could lead to bias. We have added this to the implication for future research.<br><br>Please see page 24, lines 734-736:<br><br>Future research should also include predictive variables in multiple imputation to reduce potential bias.
Supplementary table 3. What does " Adol_724" refer to? It could be clearer with a plain text name.	Thank you for your comment.<br><br>We have now replaced Adol_724 with Adolescent_724. Please see supplementary table 3.
Line 266: How many bootstraps were performed?	Thank you for your comment. We have now specified that1000 bootstraps were performed.<br><br>Please see page 12, lines 354-356:<br><br>Non-parametric bootstrapping (1000 bootstraps) with 95% confidence intervals was performed to assess the stability of the edge weights, and to determine associations that were stable and those that should be interpreted with care 13.

Results:<br><br>7. The results could be strengthened by reporting the key statistics directly in-text, e.g. most prevalent experiences, weights of the strongest edges, so that readers can follow the key findings without referring to supplementary tables/figures. This would also help as readers may not recall what specific criteria "strong/moderate/weak" refer to	Thank you for your comment. We have reported key statistics e.g., most prevalent experiences, weights of the strongest edges in the manuscript.<br><br>Please see page 14, lines 416-421:<br><br>For both adolescents and adults, living in disorganised neighbourhood was the most prevalent adverse experience (adolescents, n =753; adults, n = 490 adults, depressive symptoms was the most prevalent mental health symptoms (adolescents, n = 356; adults, n = 232), and professional help-seeking was the most prevalent form of help-seeking (adolescents, n = 213; adults, n = 185).<br><br>Please see page 15, lines 426-432:<br><br>In both adolescent and adult networks, we found a strong and stable association between AE3 “living in disorganised neighbourhood” and AE4 “living in incohesive neighbourhood” (r = 1.53, adolescents; r = 1.63, adolescents), MH1 “depressive symptoms” and MH2 “anxiety symptoms” (r = 1.95, adolescents; r = 1.72, adults), and HS1 “professional help-seeking” and HS2 “spiritual help-seeking” (r = 3.05, adolescents; r = 2.69 adults);

VERSION 2 - REVIEW

**Reviewer**	**1**
**Name**	**De Vries, Tjeerd Rudmer**
**Affiliation**	**Copenhagen Health Complexity Center**
**Date**	**21-Sep-2026**
**COI**	**NA**

Thank you very much for the revised version of this very interesting and important manuscript. The authors have satisfactorily addressed all the issues I raised previously. I have one minor comment left:

I think the discussion about centrality metrics in networks is an interesting, and unresolved, discussion, so I think it is defensible to include the analysis of such metrics in the present manuscript (and elsewhere). That said, I think the current addition of the limitations of the centrality indices is better placed in the discussion section of the manuscript, and not the introduction as it is currently.

VERSION 2 - AUTHOR RESPONSE

Reviewer 1

Comments	Response
I think the discussion about centrality metrics in networks is an interesting, and unresolved, discussion, so I think it is defensible to include the analysis of such metrics in the present manuscript (and elsewhere). That said, I think the current addition of the limitations of the centrality indices is better placed in the discussion section of the manuscript, and not the introduction as it is currently.	Thank you for your comment. We have now removed the limitations of the centrality indices from the introduction to the discussion. **Please see page 20-21, lines 618-628:**<br><br>The relevance of other centrality indices, particularly betweenness and closeness, remain debated in mental health research because their interpretation is not straightforward and they have been shown to be unstable in some cross-sectional networks.[12,63] Nevertheless, these indices may complement the strength centrality by capturing other aspects of a node's position within the networks.[63] In the adolescent network, family history of mental illness and substance use behaviour had the highest betweenness, while violence victimisation had the highest closeness. In the adult network, spiritual help-seeking had the highest betweenness and closeness. The high betweenness and closeness of these nodes may help generate hypothesis about the processes underlying the network structure of adverse experiences, mental health symptoms, and help-seeking among adolescents and adults.[63] However, these findings should be interpreted cautiously.
