# Supplementary material for: Network analysis of association and clustering of adverse experiences, mental health symptoms and help-seeking among adolescents and adults in Ghana
Source: BMJ Open. 2026 Nov 9;16(11):e121157. doi: 10.1136/bmjopen-2026-121157 (PMC13756331; doi:10.1136/bmjopen-2026-121157)
Supplement: Supplementary file 2 [file bmjopen-16-11-s001.docx]

**Supplementary Materials**

[Supplementary Table 1. Strengthening the reporting of observational studies in epidemiology reporting checklist	2](#_Toc238229255)

[Supplementary Table 2. Measures of adverse experiences	5](#_Toc238229256)

[Supplementary Table 3. Missingness at variable level (multiple imputation report)	6](#_Toc238229257)

[Supplementary Table 4. Listwise data loss report	8](#_Toc238229258)

[Supplementary Table 5. Descriptive statistics	9](#_Toc238229259)

[Supplementary Table 6. Edge weight values in adolescent network	11](#_Toc238229260)

[Supplementary Table 7. Edge weight values in adult network	12](#_Toc238229261)

[Supplementary Table 8. Results of edge invariance test	13](#_Toc238229262)

[Supplementary Figure 1. Nonparametric bootstrapped 95% confidence intervals of estimated edge weights in adolescent network (y = 0.25; OR edge rule)	16](#_Toc238229263)

[Supplementary Figure 2. Nonparametric bootstrapped 95% confidence intervals of estimated edge weights in adult network (y = 0.25; OR edge rule rule)	17](#_Toc238229264)

[Supplementary Figure 3. Sensitivity analysis result for adolescent and adult networks re-estimated using AND rule (y = 0.25)	18](#_Toc238229265)

[Supplementary Figure 4. Sensitivity analysis result for adolescent and adult networks re-estimated using y = 0.00 (OR edge rule)	19](#_Toc238229266)

[Supplementary Figure 5. Edge weights in adolescent and adult networks (y = 0.00; OR edge rule)	20](#_Toc238229267)

[Supplementary Figure 6. Edge weights in adolescent network re-estimated using 724 randomly selected adolescent sample (y = 0.25; OR edge rule)	21](#_Toc238229268)

[Supplementary Figure 7. Sensitivity analysis result for adolescent and adult network re-estimated using listwise deletion method	22](#_Toc238229269)

[Supplementary Figure 8. Edge weights in adolescent and adult network re-estimated using listwise deletion method	23](#_Toc238229270)

[Supplementary Figure 9. Centrality plot of betweenness	24](#_Toc238229271)

[Note:	24](#_Toc238229272)

[Supplementary Figure 10. Centrality plot for closeness	24](#_Toc238229273)

[Supplementary Figure 11. Centrality stability plots for adolescents and adults.	25](#_Toc238229274)

## Supplementary Table 1. Strengthening the reporting of observational studies in epidemiology reporting checklist

|  | | **Item No** | **Recommendation** | **Page number** |
| --- | --- | --- | --- | --- |
| **Title and abstract** | | 1 | (*a*) Indicate the study’s design with a commonly used term in the title or the abstract | Pg. 1 |
|  |  |  | (*b*) Provide in the abstract an informative and balanced summary of what was done and what was found | Pg. 2-3 |
| **Introduction** | | | |  |
| Background/rationale | | 2 | Explain the scientific background and rationale for the investigation being reported | Pg. 4-7 |
| Objectives | | 3 | State specific objectives, including any prespecified hypotheses | Pg. 7 |
| **Methods** | | | |  |
| Study design | | 4 | Present key elements of study design early in the paper | Pg. 7 |
| Setting | | 5 | Describe the setting, locations, and relevant dates, including periods of recruitment, exposure, follow-up, and data collection | Pg. 7 |
| Participants | | 6 | (*a*) *Cohort study*—Give the eligibility criteria, and the sources and methods of selection of participants. Describe methods of follow-up  *Case-control study*—Give the eligibility criteria, and the sources and methods of case ascertainment and control selection. Give the rationale for the choice of cases and controls  *Cross-sectional study*—Give the eligibility criteria, and the sources and methods of selection of participants | Pg. 8 |
|  |  |  | (*b*) *Cohort study*—For matched studies, give matching criteria and number of exposed and unexposed  *Case-control study*—For matched studies, give matching criteria and the number of controls per case | N/A |
| Variables | | 7 | Clearly define all outcomes, exposures, predictors, potential confounders, and effect modifiers. Give diagnostic criteria, if applicable | Pg. 8 & 9 |
| Data sources/ measurement | | 8* | For each variable of interest, give sources of data and details of methods of assessment (measurement). Describe comparability of assessment methods if there is more than one group | Pg. 8-9 & Supplementary Table 2 |
| Bias | | 9 | Describe any efforts to address potential sources of bias | Pg. 12 & 13 |
| Study size | | 10 | Explain how the study size was arrived at | Pg. 8 |
| Quantitative variables | | 11 | Explain how quantitative variables were handled in the analyses. If applicable, describe which groupings were chosen and why | Pg. 11-14 |
| Statistical methods | | 12 | (*a*) Describe all statistical methods, including those used to control for confounding | Pg. 11-14 |
|  |  |  | (*b*) Describe any methods used to examine subgroups and interactions | Pg. 11-14 |
|  |  |  | (*c*) Explain how missing data were addressed | Pg. 11 &12 |
|  |  |  | (*d*) *Cohort study*—If applicable, explain how loss to follow-up was addressed  *Case-control study*—If applicable, explain how matching of cases and controls was addressed  *Cross-sectional study*—If applicable, describe analytical methods taking account of sampling strategy | N/A |
|  |  |  | (*e*) Describe any sensitivity analyses | Pg. 12 |
| **Results** | | | |  |
| Participants | 13* | (a) Report numbers of individuals at each stage of study—eg numbers potentially eligible, examined for eligibility, confirmed eligible, included in the study, completing follow-up, and analysed | | Pg. 14-15 |
|  |  | (b) Give reasons for non-participation at each stage | | N/A |
|  |  | (c) Consider use of a flow diagram | | N/A |
| Descriptive data | 14* | (a) Give characteristics of study participants (eg demographic, clinical, social) and information on exposures and potential confounders | | Pg. 14 & supplementary table 5 |
|  |  | (b) Indicate number of participants with missing data for each variable of interest | | Supplementary table 3 & 4 |
|  |  | (c) *Cohort study*—Summarise follow-up time (eg, average and total amount) | | N/A |
| Outcome data | 15* | *Cohort study*—Report numbers of outcome events or summary measures over time | | N/A |
|  |  | *Case-control study—*Report numbers in each exposure category, or summary measures of exposure | | N/A |
|  |  | *Cross-sectional study—*Report numbers of outcome events or summary measures | | Pg. 14 & supplementary table 5 |
| Main results | 16 | (*a*) Give unadjusted estimates and, if applicable, confounder-adjusted estimates and their precision (eg, 95% confidence interval). Make clear which confounders were adjusted for and why they were included | | Pg. 14-18 |
|  |  | (*b*) Report category boundaries when continuous variables were categorized | | N/A |
|  |  | (*c*) If relevant, consider translating estimates of relative risk into absolute risk for a meaningful time period | | N/A |
| Other analyses | 17 | Report other analyses done—eg analyses of subgroups and interactions, and sensitivity analyses | | Pg. 15-17 |
| **Discussion** | | | |  |
| Key results | 18 | Summarise key results with reference to study objectives | | Pg. 18-22 |
| Limitations | 19 | Discuss limitations of the study, taking into account sources of potential bias or imprecision. Discuss both direction and magnitude of any potential bias | | Pg. 22-23 |
| Interpretation | 20 | Give a cautious overall interpretation of results considering objectives, limitations, multiplicity of analyses, results from similar studies, and other relevant evidence | | Pg. 18-24 |
| Generalisability | 21 | Discuss the generalisability (external validity) of the study results | | Pg. 22 |
| **Other information** | | | |  |
| Funding | 22 | Give the source of funding and the role of the funders for the present study and, if applicable, for the original study on which the present article is based | | Pg. 31 |

Note Pg. = page and N/A = not applicable

## Supplementary Table 2. Measures of adverse experiences

| Items | Indicators | Response type |
| --- | --- | --- |
| Accident involvement | I was involved in an accident or someone hit my head with an object or an object fell on my head), | Categorical (Yes/No) |
| Family history of mental illness | illness (do you know a relative or family member who suffers from mental illness?), | Categorical (Yes/No, I don’t know) |
| Living in a disorganised neighbourhood | people in my neighbourhood do not respect rules and laws | Categorical (Yes/No) |
| Living in an incohesive neighbourhood | people do not care about what goes on in the neighbourhood) | Categorical (Yes/No) |
| Physical violence victimisation | how often you been physically attacked or beaten in the last 12 months; hereinafter violence victimisation | Categorical (Yes/No); ordinal: never, occasionally, nearly always, often |
| School stress | how much stress do you usually feel because of school activities such as attending classes | Categorical (Yes/No)  Ordinal: a lot, a little bit, very much, rarely, very much |
| Substance use behaviour | have you ever smoked or used alcohol | Categorical (Yes/No) ordinal: never, occasionally, nearly always, often |

## Supplementary Table 3. Missingness at variable level (multiple imputation report)

| Dataset | Variable | Missing_Count | Missing_Percent |
| --- | --- | --- | --- |
| All | Depressive symptoms | 0 | 0 |
| All | Anxiety symptoms | 0 | 0 |
| All | Accident involvement | 13 | 0.69 |
| All | Violence victimisation | 10 | 0.53 |
| All | Substance use behaviour | 0 | 0 |
| All | Family history of mental illness | 615 | 32.64 |
| All | Disorganised neighbourhood | 41 | 2.18 |
| All | Incohesive neighbourhood | 43 | 2.28 |
| All | School stress | 8 | 0.42 |
| All | Professional help-seeking | 0 | 0 |
| All | Spiritual help-seeking | 0 | 0 |
| Adolescent | Depressive symptoms | 0 | 0 |
| Adolescent | Anxiety symptoms | 0 | 0 |
| Adolescent | Accident involvement | 7 | 0.6 |
| Adolescent | Violence victimisation | 6 | 0.52 |
| Adolescent | Substance use behaviour | 0 | 0 |
| Adolescent | Family history of mental illness | 399 | 34.4 |
| Adolescent | Disorganised neighbourhood | 20 | 1.72 |
| Adolescent | Incohesive neighbourhood | 22 | 1.9 |
| Adolescent | School stress | 4 | 0.34 |
| Adolescent | Professional help-seeking | 0 | 0 |
| Adolescent | Spiritual help-seeking | 0 | 0 |
| Adult | Depressive symptoms | 0 | 0 |
| Adult | Anxiety symptoms | 0 | 0 |
| Adult | Accident involvement | 6 | 0.83 |
| Adult | Physical violence victimisation | 4 | 0.55 |
| Adult | Substance use behaviour | 0 | 0 |
| Adult | Family history of mental illness | 216 | 29.83 |
| Adult | Disorganised neighbourhood | 21 | 2.9 |
| Adult | Incohesive neighbourhood | 21 | 2.9 |
| Adult | School stress | 4 | 0.55 |
| Adult | Professional help-seeking | 0 | 0 |
| Adult | Spiritual help-seeking | 0 | 0 |
| Adolescent_724 | Depressive symptoms | 0 | 0 |
| Adolescent_724 | Anxiety symptoms | 0 | 0 |
| Adolescent_724 | Accident involvement | 3 | 0.41 |
| Adolescent_724 | Violence victimisation | 1 | 0.14 |
| Adolescent_724 | Substance use behaviour | 0 | 0 |
| Adolescent_724 | Family history of mental illness | 252 | 34.81 |
| Adolescent_724 | Disorganised neighbourhood | 14 | 1.93 |
| Adolescent_724 | Incohesive neighbourhood | 15 | 2.07 |
| Adolescent_724 | School stress | 1 | 0.14 |
| Adolescent_724 | Professional help-seeking | 0 | 0 |
| Adolescent_724 | Spiritual help-seeking | 0 | 0 |
| Percentage summary of imputed data | | | |
| Adolescent | 3.52 | | |
| Adult | 3.42 | | |
| Adol_724 | 3.79 | | |

## Supplementary Table 4. Listwise data loss report

| Dataset | Total_Cases | Retained_Cases | Lost_Cases | Percent_Lost |
| --- | --- | --- | --- | --- |
| Adolescent | 1160 | 739 | 421 | 36.29 |
| Adult | 724 | 480 | 244 | 33.7 |
| Adolescent boys | 380 | 238 | 142 | 37.37 |
| Adolescent girls | 780 | 501 | 279 | 35.77 |
| Adult men | 332 | 222 | 110 | 33.13 |
| Adult women | 392 | 258 | 134 | 34.18 |

## Supplementary Table 5. Descriptive statistics

| Demographic | Overall sample  (n = 1884) | Adolescents 12-17 years  (n = 1160) | Adults  18-45 years (n = 724) |
| --- | --- | --- | --- |
| Age; mean (SD) | 17.3 (1.62) | 16.4 (0.76) | 18.7 (1.62) |
| Gender | | | |
| Boys/Men | 712 (37.8%) | 380 (32.8%) | 332 (45.9%) |
| Girls/Women | 1172 (62.2%) | 780 (67.2%) | 392 (54.1%) |
| Missing data | 0 | 0 | 0 |
| Area of residence | | | |
| Rural | 518 (27.9%) | 256 (22.4%) | 262 (36.8%) |
| Urban | 778 (41.9%) | 548 (47.9%) | 230 (32.3%) |
| Peri-urban | 561 (30.2%) | 341 (29.8%) | 220 (30.9%) |
| Missing data |  |  |  |
| Physical activity | | | |
| 0 days | 798 (62.5%) | 475 (60.4%) | 323 (65.8%) |
| 1-3 days | 243 (19.0%) | 150 (19.1%) | 93 (18.9%) |
| Above 7 days | 236 (18.5%) | 161 (20.5%) | 75 (15.3%) |
| Missing data | 607 | 374 | 233 |
| Adverse experiences | | | |
| Accident involvement | | | |
| Yes | 624 (33.4%) | 374 (32.4%) | 250 (34.8%) |
| No | 1247 (66.6%) | 779 (67.6%) | 468 (65.2%) |
| Missing data | 13 |  |  |
| Family history of mental illness | | | |
| Yes | 273 (21.5%) | 615 (80.8%) | 381 (75.0%) |
| No | 996 (78.5%) | 146 (19.2%) | 127 (25.0%) |
| Missing data | 615 | 399 | 216 |
| Living in disorganised neighbourhood | | | |
| Yes | 1243 (67.4%) | 753 (66.1%) | 490 (69.7%) |
| No | 600 (32.6%) | 387 (33.9%) | 213 (30.3%) |
| Missing data | 41 | 20 | 21 |
| Living in incohesive neighbourhood | | | |
| Yes | 1147 (62.3%) | 674 (59.2%) | 473 (67.3%) |
| No | 694 (37.7%) | 464 (40.8%) | 230 (32.7%) |
| Missing data | 43 | 22 | 21 |
| Violence victimisation | | | |
| Yes | 489 (26.1%) | 282 (24.4%) | 207 (28.7%) |
| No | 1385 (73.9%) | 872 (75.6%) | 513 (71.2%) |
| Missing data | 10 | 6 | 4 |
| School Stress | | | |
| Yes | 875 (46.6%) | 523 (45.2%) | 352 (48.9%) |
| No | 1001 (53.4%) | 633 (54.8%) | 368 (51.1%) |
| Missing data | 8 | 4 | 4 |
| Substance use behaviour |  |  |  |
| Yes | 316 (16.8%) | 182 (15.7%) | 134 (18.5%) |
| No | 1568 (83.2%) | 978 (84.3%) | 590 (81.5%) |
| Missing data | 0 | 0 | 0 |
| Mental health symptoms |  |  |  |
| Depressive symptoms (PHQ-9 > 10) | 588 (31.2%) | 356 (30.7%) | 232 (32%) |
| Anxiety symptoms (GAD-7 > 10) | 311 (16.5%) | 195 (16.8%) | 116 (16%) |
| Missing data | - | - | - |
| Help-seeking | | | |
| Professional help-seeking | | | |
| Yes | 398 (21.1%) | 213 (18.4%) | 185 (25.6%) |
| No | 1486 (78.9%) | 947 (81.6%) | 539 (74.4%) |
| Missing data | - | - | - |
| Spiritual help-seeking | | | |
| Yes | 337 (17.9%) | 182 (15.7%) | 155 (21.4%) |
| No | 1547 (82.1%) | 978 (84.3%) | 569 (78.6%) |
| Missing data | - | - | - |

***Note.*** Family history of with mental illness was based on n of 1271

## Supplementary Table 6. Edge weight values in adolescent network

| Node 1 | Node 2 | Edge weight |
| --- | --- | --- |
| Depressive symptoms | Anxiety symptoms | 1.95 |
| Depressive symptoms | Violence victimisation | 0.47 |
| Depressive symptoms | Substance use behaviour | 0.30 |
| Depressive symptoms | Disorganised neighbourhood | 0.04 |
| Depressive symptoms | School stress | 0.46 |
| Anxiety symptoms | Family history of mental illness | 0.35 |
| Accident involvement | Substance use behaviour | 0.31 |
| Accident involvement | Family history of mental illness | 0.14 |
| Violence victimisation | Substance use behaviour | 0.47 |
| Violence victimisation | Family history of mental illness | 0.44 |
| Violence victimisation | School stress | 0.24 |
| Substance use behaviour | Disorganised neighbourhood | 0.24 |
| Family history of mental illness | Spiritual help-seeking | 0.08 |
| Disorganised neighbourhood | Incohesive neighbourhood | 1.53 |
| Professional help-seeking | Spiritual help-seeking | 3.05 |

*Note:* In the adolescent network, the strength of the association between accident involvement and substance use behaviour, depressive symptoms and violence victimisation, and depressive symptoms and substance use behaviour, was moderate. In contrast, the associations between depressive symptoms and living in a disorganised neighbourhood, family history of mental illness and spiritual help‑seeking, and violence victimisation and school stress, respectively, was weak.

## Supplementary Table 7. Edge weight values in adult network

| Node 1 (From) | Node 2 (To) | Edge weight |
| --- | --- | --- |
| Depressive symptoms | Anxiety symptoms | 1.72 |
| Depressive symptoms | Violence victimisation | 0.33 |
| Depressive symptoms | Spiritual help-seeking | 0.34 |
| Physical violence victimisation | Family history of mental illness | 0.06 |
| Family history of mental illness | Disorganised neighbourhood | 0.22 |
| Family history of mental illness | Spiritual help-seeking | 0.19 |
| Disorganised neighbourhood | Incohesive neighbourhood | 1.63 |
| Professional help-seeking | Spiritual help-seeking | 2.69 |

*Note:* In the adult network, the associations between depressive symptoms and violence victimisation, and depressive symptoms and spiritual help‑seeking were moderate, whereas the associations between family history of mental illness and living in a disorganised neighbourhood, family history of mental illness and spiritual help‑seeking, and violence victimisation and family history of mental illness were weak. Across both networks, these associations were unstable across bootstrapped resampling and should be confirmed in future studies.

## Supplementary Table 8. Results of edge invariance test

| Edge index | edge | P_value | Significance |
| --- | --- | --- | --- |
| 1. | Depressive symptoms and accident involvement | 0 | true |
| 2. | Anxiety symptoms and accident involvement | 0 | true |
| 3. | Depressive symptoms and violence victimisation | 0.322606 | false |
| 4. | Anxiety symptoms and violence victimisation | 0 | true |
| 5. | Accident involvement and violence victimisation | 0 | true |
| 6. | Depressive symptoms and substance use behavior | 0 | true |
| 7. | anxiety symptoms and substance use behaviour | 0 | True |
| 8. | Accident involvement and substance use behaviour | 0 | True |
| 9. | Violence victimisation and substance use behaviour | 0 | True |
| 10. | Depressive symptoms and family history of mental illness | 0 | True |
| 11. | Anxiety symptoms and family history of mental illness | 0 | True |
| 12. | Accident involvement and family history of mental illness | 0 | True |
| 13. | Violence victimisation and family history of mental illness | 0 | True |
| 14. | Substance use behaviour and family history of mental illness | 0 | True |
| 15. | Depressive symptoms and living in disorganised neighbourhood | 0 | True |
| 16. | Anxiety symptoms and living in disorganised neighbourhood | 0 | True |
| 17. | Accident involvement and living in disorganised neighbourhood | 0 | True |
| 18. | Violence victimisation and living in disorganised neighbourhood | 0 | True |
| 19. | Substance use behaviour and living in disorganised neighbourhood | 0 | True |
| 20. | Family history of mental illness and living in disorganised neighbourhood | 0 | True |
| 21. | Depressive symptoms and living in incohesive neighbourhood | 0 | True |
| 22. | Anxiety symptoms and living in incohesive neighbourhood | 0 | True |
| 23. | Accident involvement and living in incohesive neighbourhood | 0 | True |
| 24. | Violence victimisation and living in incohesive neighbourhood | 0 | True |
| 25. | Substance use behaviour and living in incohesive neighbourhood | 0 | True |
| 26. | Family history of mental illness and living in incohesive neighbourhood | 0 | True |
| 27. | Living in disorganised neighbourhood and living in incohesive neighbourhood | 0.286848 | False |
| 28. | Depressive symptoms and school stress | 0.32774 | False |
| 29. | Anxiety symptoms and school stress | 0 | True |
| 30. | Accident involvement and school stress | 0 | True |
| 31. | Violence victimisation and school stress | 0 | True |
| 32. | Substance use behaviour and school stress | 0 | True |
| 33. | Family history of mental illness and school stress | 0 | True |
| 34. | Living in disorganised neighbourhood and school stress | 0 | True |
| 35. | Living in incohesive neighbourhood and school stress | 0 | True |
| 36. | Depressive symptoms and professional help-seeking | 0 | True |
| 37. | Anxiety symptoms and professional help-seeking | 0 | True |
| 38. | Accident involvement and professional help-seeking | 0 | True |
| 39. | Violence victimisation and professional help-seeking | 0 | True |
| 40. | Substance use behaviour and professional help-seeking | 0 | True |
| 41. | Family history of mental illness and professional help-seeking | 0 | True |
| 42. | Living in disorganised neighbourhood and professional help-seeking | 0 | True |
| 43. | Living in incohesive neighbourhood and professional help-seeking | 0 | True |
| 44. | School stress and professional help-seeking | 0 | True |
| 45. | Depressive symptoms and spiritual help-seeking | 0.381605 | False |
| 46. | Anxiety symptoms and spiritual help-seeking | 0 | True |
| 47. | Accident involvement and spiritual help-seeking | 0 | True |
| 48. | Violence victimisation and spiritual help-seeking | 0 | True |
| 49. | Substance use behaviour and spiritual help-seeking | 0 | True |
| 50. | Family history of mental illness and spiritual help-seeking | 0.448883 | False |
| 51. | Living in disorganised neighbourhood and spiritual help-seeking | 0 | True |
| 52. | Living in incohesive neighbourhood and spiritual help-seeking | 0 | True |
| 53. | School stress and spiritual help-seeking | 0 | True |
| 54. | Professional help-seeking and spiritual help-seeking | 0.292491 | False |

## Supplementary Figure 1. Nonparametric bootstrapped 95% confidence intervals of estimated edge weights in adolescent network (y = 0.25; OR edge rule)

## Supplementary Figure 2. Nonparametric bootstrapped 95% confidence intervals of estimated edge weights in adult network (y = 0.25; OR edge rule rule)

## Supplementary Figure 3. Sensitivity analysis result for adolescent and adult networks re-estimated using AND rule (y = 0.25)

## Supplementary Figure 4. Sensitivity analysis result for adolescent and adult networks re-estimated using y = 0.00 (OR edge rule)

## Supplementary Figure 5. Edge weights in adolescent and adult networks (y = 0.00; OR edge rule)

## Supplementary Figure 6. Edge weights in adolescent network re-estimated using 724 randomly selected adolescent sample (y = 0.25; OR edge rule)

## Supplementary Figure 7. Sensitivity analysis result for adolescent and adult network re-estimated using listwise deletion method

## Supplementary Figure 8. Edge weights in adolescent and adult network re-estimated using listwise deletion method

## Supplementary Figure 9. Centrality plot of betweenness

Note: this bar plot illustrates the betweenness value of each node in the adolescent and adult networks. The y-axis represents the centrality indices quantified in z-scores. Higher values indicate higher betweenness, while lower values represent lower betweenness.

## Supplementary Figure 10. Centrality plot for closeness

Note: this bar plot illustrates the closeness value of each node in the adolescent and adult networks. The y-axis represents the centrality indices quantified in z-scores. Higher values indicate higher closeness, while lower values represent lower closeness.

## Supplementary Figure 11. Centrality stability plots for adolescents and adults.

*Note*: The x-axis represents the percentage of original sample used at each step. The y-axis represents the average of correlations between the centrality indices from the original network and the centrality indices from the networks that were re-estimated after the case dropping procedure.
